# Tetrakis-Cyanoacetylides as Building Blocks for a Second Generation of Spin-Switchable Hofmann-type Networks with Enhanced Porosity

**DOI:** 10.1021/acs.inorgchem.4c02732

**Published:** 2024-09-03

**Authors:** Willi Zeni, Danny Müller, Werner Artner, Gerald Giester, Michael Reissner, Peter Weinberger

**Affiliations:** † Institute of Applied Synthetic Chemistry, TU Wien, Getreidemarkt 9/163-01-3, 1060 Vienna, Austria; ‡ X-Ray Center, TU Wien, Getreidemarkt 9/057-4, 1060 Vienna, Austria; § Department of Mineralogy and Crystallography, University of Vienna, Josef-Holaubek-Platz 2, 1090 Vienna, Austria; ∥ Institute of Solid State Physics, TU Wien, Wiedner Hauptstraße 8-10/138, 1040 Vienna, Austria

## Abstract

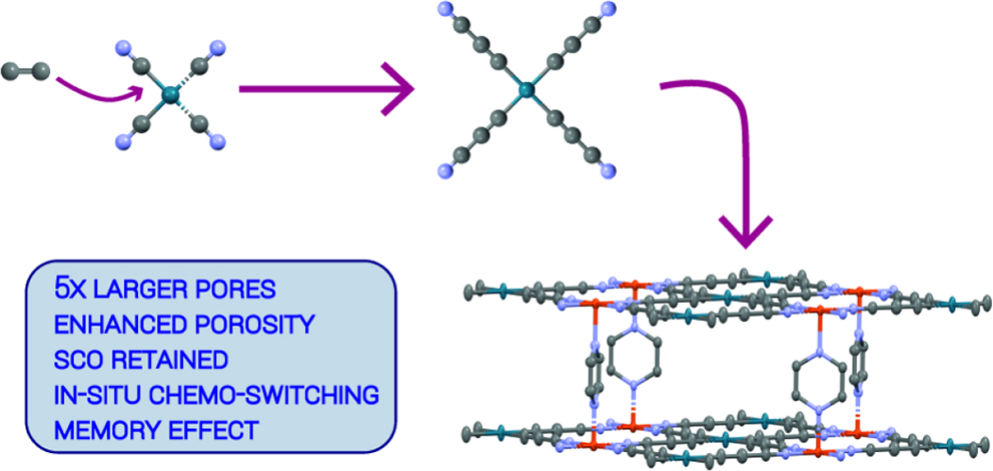

The combination of spin crossover (SCO) with guest incorporation properties has attracted the interest of researchers in the last couple of decades and has led to the design of numerous SCO porous coordination polymers (SCO-PCPs). The most famous class of SCO-PCPs is the Hofmann-type network, which is a very promising material for (chemo)sensing applications. Different strategies have been carried out to expand the classic structure {Fe(pz)[M^II^(CN)_4_]} (M = Ni, Pd, Pt) to get larger cavities, but the resulting compounds often showed a poor magnetic behavior. In this work, we present wide-mesh-size spin-switching Hofmann-type networks based on tetrakis-cyanoacetylides synthesized with a newly developed method, resulting in compounds with the general formula {Fe(pz)[M(C_3_N)_4_]} (M=Ni, Pd, Pt). The compounds were characterized in their structural, magnetic, and spectroscopic properties. They present 5-fold larger cavities and a drastic increase in porosity. The desired hysteretic and guest-dependent spin-crossover behavior is retained, and in situ chemo-switching of the spin state and the memory effect are also observed.

## Abstract 2

A newly developed synthetic method was used to prepare spin-crossover Hofmann-type PCPs with enhanced porosity. Instead of using longer pillar ligands, the elongation was performed on the *ab* plane, using tetrakis-cyanoacetylide linkers instead of classic tetracyanometallates. The compounds retained the desired 3D open framework structure, with a 5-fold increase of the pores’ volume and a notable increase in porosity. Guest-dependent spin transition, in situ chemo-switching, and the memory effect were observed.

## Introduction

Porous coordination polymers (PCPs) gained considerable interest in the last couple decades as a class of porous materials which, thanks to their permanent and designable regular porosity, showed great versatility and created prospects for their application in different fields, from hydrogen storage,^1^ natural gas storage,^2^ gas separation,^3−7^ and capturing of harmful gases^8^ to an application in catalysis^9^ or as a stationary phase in gas chromatography.^10^ A current research focus in the field of multifunctionality is the creation of systems in which the guest adsorption process is accompanied by a change in solid-state properties (e.g., optics, conductivity, magnetism). In this context, the coupling of the porous properties with the spin crossover (SCO) phenomenon is particularly attractive, especially since in the last two decades the research in the SCO field has been focused on multifunctional materials. SCO is well known for Fe(II) coordination compounds, showing spin-state interconversions between the low-spin (LS) and high-spin (HS) states under external stimuli (temperature,^11,12^ pressure,^11−13^ light,^14,15^ etc.). The change of the metal center’s spin state affects key properties of the material including the magnetic moment,^11,12^ color,^16^ dielectric constant,^17,18^ lattice extension,^19^ etc. The most famous class of spin-switchable porous networks is the one of Hofmann-type networks, based on the compound M1(L)_2_[M2(CN)_4_] (M1 = M2 = Ni^2+^; L = NH_3_) reported in 1897 by Hofmann and Küspert.^20^ The first SCO-PCP, namely, the two-dimensional (2D) PCP Fe(py)_2_[Ni(CN)_4_] (py = pyridine), was reported in 1996 by Kitazawa et al.^21^ and represented the first milestone in SCO-PCP research. In 2001, the second milestone was reached, with {Fe(pz)[M^II^(CN)4]} (pz = pyrazine, M^II^ = Ni, Pd, Pt) reported by Real et al.^22^ The substitution of py with pz resulted in {Fe[M^II^(CN)_4_]∞} layers stacked by pz ligands, giving the network a three-dimensional (3D) dimensionality. Since then, this class of compounds rapidly gained interest for a variety of reasons: (a) the highly cooperative and often hysteretic spin transition around room temperature; (b) the guest-responsive SCO, guest-dependent magnetic behavior, and memory effect; (c) robustness to absorption and desorption of a wide range of small-molecule guests; and (d) the possibility to tailor the design of the frameworks, making these systems very flexible and adaptable to the desired application.

In 2009, Ohba et al. published a study in which they reported the bidirectional chemo-switching of the spin state in {Fe(pz)[Pt(CN)_4_]} (=**pzPt**), demonstrating how this class of PCPs are truly environmentally responsive materials. Furthermore, the presence of the memory effect was observed, for which the guest-induced spin state was retained even after guest desorption.^23^ A synergistic interplay between SCO and guest exchange was also demonstrated for {Fe(pz)[Ni(CN)_4_]} (=**pzNi**) by Kepert et al.^24^

Numerous modifications of the {Fe(pz)[M^II^(CN)_4_]} (=**pzM**) scaffold have been reported, mainly with the goal of increasing the pores’ volume and enhancing the porosity of the PCPs, which would allow for the incorporation of larger guest molecules, instead of only small, passive molecules. The extension of the pores’ size has been mostly achieved by substituting pz with longer ditopic N-ligands, such as bpac^25^ or bpeben^26^ (bpac = bis(4-pyridyl)acetylene, bpeben = 1,4-bis(4-pyridylethynyl)benzene). While the desired 3D structure was preserved and the pores’ volume was indeed increased (10 times increase in the case of bpeben compared to pz), these compounds showed a gradual and nonhysteretic spin transition, suggesting that the effectiveness of the transmission of SCO cooperativity decreases with the increasing length of the pillar ligand. Furthermore, the incorporation of the ligand in the pores was observed. Another strategy to increase the pores’ volume is the substitution of the [M^II^(CN)_4_]^2–^ linkers with [M^I^(CN)_2_]^−^ (M^I^ = Ag, Cu, Au); the use of [Ag(CN)_2_]^−^ yields [Fe(L)*_n_*{Ag(CN)_2_}_2_], with a structure consisting of two interpenetrating 3D networks with edge-shared {Fe[Ag(CN)_2_]_4_} rhombuses in the 2D sheets.^27^ In the case of Au, 3D triply interpenetrated networks are formed.^28−30^ This approach successfully enlarged the voids so that even a ferrocene molecule could be incorporated,^30^ but again, the compounds showed a poor magnetic behavior. Furthermore, network interpenetration takes away the regular porosity, making the system less flexible for possible modifications.

In this work, we present a previously unreported method for the synthesis of a new generation of expanded Hofmann-type networks. The elongation is performed by the insertion of an acetylenic subunit to the M–CN bond, resulting in a square-planar tetrakis-cyanoacetylide [M^II^(C_3_N)_4_]^2–^ spacer with a nearly doubled extension compared to [M^II^(CN)_4_]^2–^, leading to Hofmann-type SCO-PCPs of the general formula {Fe(pz)[M^II^(C_3_N)_4_]}. The absence of additional metallic nodes preserves the desired 3D open framework structure with regular porosity avoiding networks’ interpenetration. The magnetic properties are retained featuring hysteretic SCO near room temperature, as well as the guest dependency of the spin state, guest-induced spin transition (chemo-switching), and the memory effect shown by {Fe(pz)[M^II^(CN)_4_]}, but in comparison, the here reported compounds feature 5-fold larger pores and a drastic increase in porosity.

## Results and Discussion

### Synthesis

**Scheme 1:**
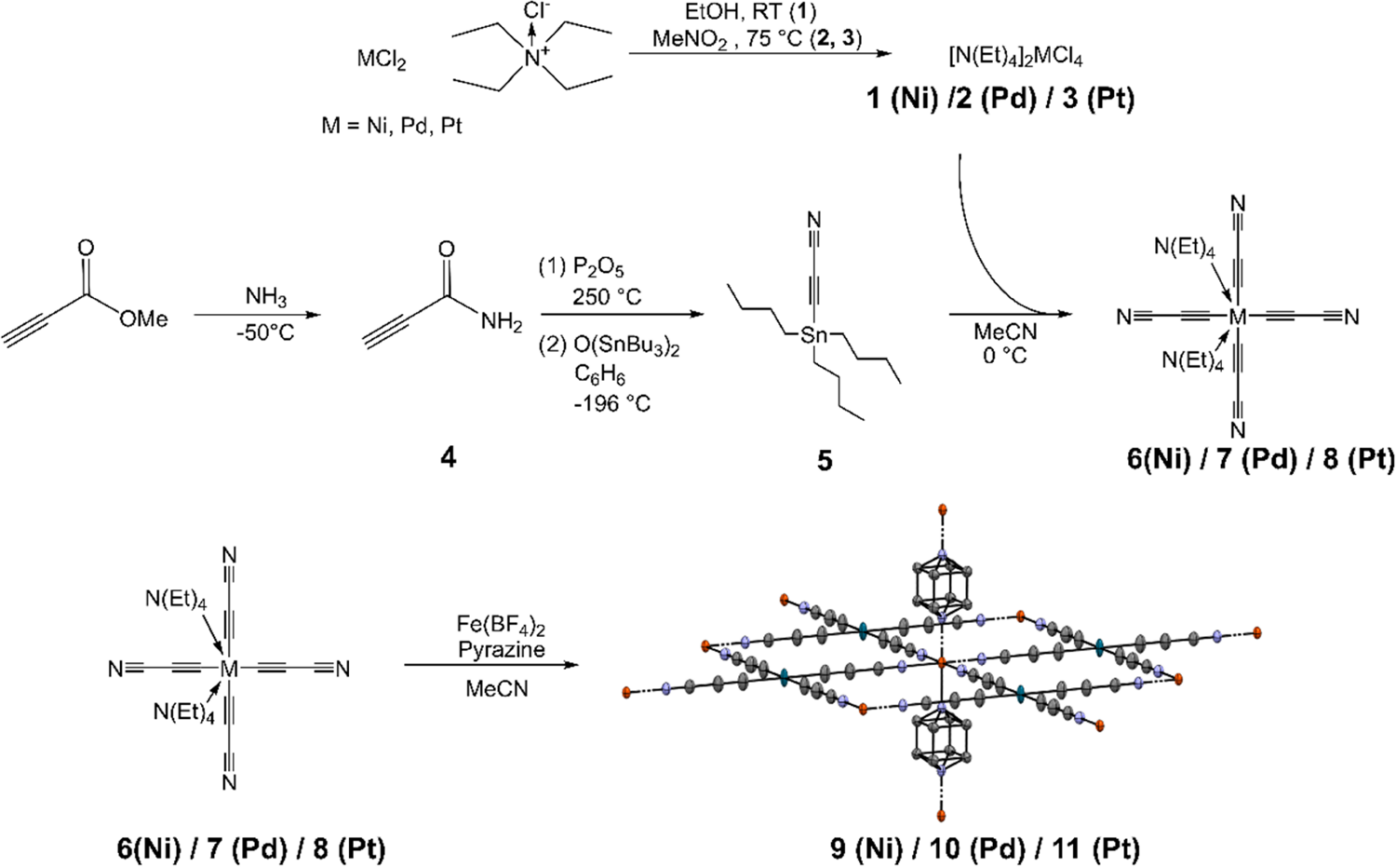
Synthetic Route for the Preparation of Fe(pz)[M(C_3_N)_4_]

The preparation of the [M^II^(C_3_N)_4_]^2–^ species (M = Ni, Pd, Pt) was achieved with a slight modification of a literature-known procedure,^31^ where instead of Me_3_SnC_3_N, the tributyl analogue *n*Bu_3_SnC_3_N was used, and MeCN was used as the solvent instead of dimethylformamide (DMF). The first step consisted in the synthesis of [NEt_4_]_2_[MCl_4_] (**1/2/3** for Ni, Pd, and Pt, respectively) by the reaction of MCl_2_ with NEt_4_Cl. The synthesis of nBu_3_SnC_3_N was also done by modifying a literature-reported procedure:^32^ the reaction of methylpropiolate with NH_3_ at −50 °C led to the formation of 2-propynamide (**4**), which was first treated with phosphorus pentoxide for dehydration and subsequently with bis(tributyltin) oxide, leading to the formation of 3-(tributylstannyl)propiolonitrile (**5**). Ultimately, the reaction of **1/2/3** with **5** in MeCN at 0 °C resulted in the formation of [NEt_4_][M(C_3_N)_4_] (**6/7/8** for Ni, Pd, and Pt, respectively). As reported in literature,^31^ the compounds are air-stable in the solid state, but in contrast to literature reports, the species are stable in solution for longer than 24 h if an inert atmosphere is provided. After a longer time, a small deposit forms. The PCPs were synthesized by mixing **6**–**8** with anhydrous Fe(BF_4_)_2_ and pyrazine in MeCN, where compounds **9**, **10**, and **11** precipitated as amorphous powders. After centrifugation and solvent removal, the powders were dried in vacuum. In the HS state, the PCPs appear as yellow/orange powders, and in the LS state, the color changes to deep red. A schematic representation of the synthesis is depicted in Scheme 1; for detailed experimental procedures, see the Experimental Section.

The synthesis of **6/7/8** with the use of nBu_3_SnC_3_N allowed for lower volumes of solvents and easier precipitation of the products in Et_2_O. Strict exclusion of oxygen and moisture are crucial for a successful synthesis, especially for the more reactive compound **6**, which shows a tendency for homopolymerization of the propargylnitrile fragment when these criteria are not met. The only drawback is a somewhat difficult workup, caused by the muddy texture of the reaction mixture, which makes the filtration a bit tedious. Another suboptimal synthetic aspect, which also regards **5**, is the use of trialkyl-Sn^IV^ species, whose toxicity is well known. To avoid its use, other synthetic pathways were tried out (e.g., isolation of HC_3_N, deprotonation to form an acetylide, and subsequent reaction with **1**–**3**) but they were unsuccessful. Since organolithium reagents cannot be used due to the presence of the CN group, sodium amide was utilized as the base, but some problems were encountered: on one hand, it is very hard to find a solvent (or solvent mixture) in which NaNH_2_ is, even slightly, soluble (NH_3_ would be the best choice in that sense, but here it is inconvenient since it is also the byproduct of the reaction); on the other hand, one must deal with the very high thermal instability of acetylides. To overcome the second problem, syntheses at low temperatures (up to −90 °C) were carried out, but they were again unsuccessful since seconds after mixing the reagents, the product decomposed. Even lower temperature conditions could be tried out, but that again poses a solubility problem, and finding a solvent that is still liquid at such temperatures is also challenging. Finally, it must be mentioned that compound **9** is very temperature-sensitive and decomposes at ∼35 °C. Thermal analysis of **10** and **11** shows one step relative to the loss of one molecule of pyrazine for both **10** and **11**. Characteristic temperatures of the steps are 361 and 340 °C for **10** and **11**, respectively (see Figures S17 and S18 of the SI).

### Structural Characterization

Single crystals suitable for X-ray diffraction could solely be grown for compound **10** via electrocrystallization and could only be measured for the LS state at 200 K due to degradation of the crystals at higher temperatures. Structural confirmation for compounds **9** and **11** was obtained via powder X-ray diffraction (PXRD), exploiting the isostructurality of the PCPs and resulting in identical PXRD patterns. A layering technique with a very diluted solution was used to obtain the crystalline powder. A detailed description of the experimental setup is given in the Experimental Section.

Compound **10** may be described in the tetragonal space group *P*4/*mmm* (no. 123) with one molecule per unit cell (*a* = 10.795(2) Å, *c* = 6.759 (2) Å, and *V* = 787.6 Å^3^) with disordered pz moieties. However, taking into account the rather weak additional X-ray reflections, a superstructure results in the space group *P*4/*mbm* (no. 127) with two molecules per unit cell (*a* = 15.266 (3) Å, *c* = 6.759 (2) Å, and *V* = 1575.2 Å^3^). This now allows an ordered arrangement of the pyrazine molecules, as shown in the Supporting Figure S1. For further details, the reader is referred to the methodology chapter. For the sake of simplicity, the following further structure description will only be done in the small unit cell in the space group *P*4/*mmm*. It must also be mentioned that the cavities most likely contain acetonitrile solvate molecules: however, they are highly disordered so much so that they could not be located. Therefore, they were ignored in the refinement procedure, and the structures are presented without the solvent.

**Figure 1:**
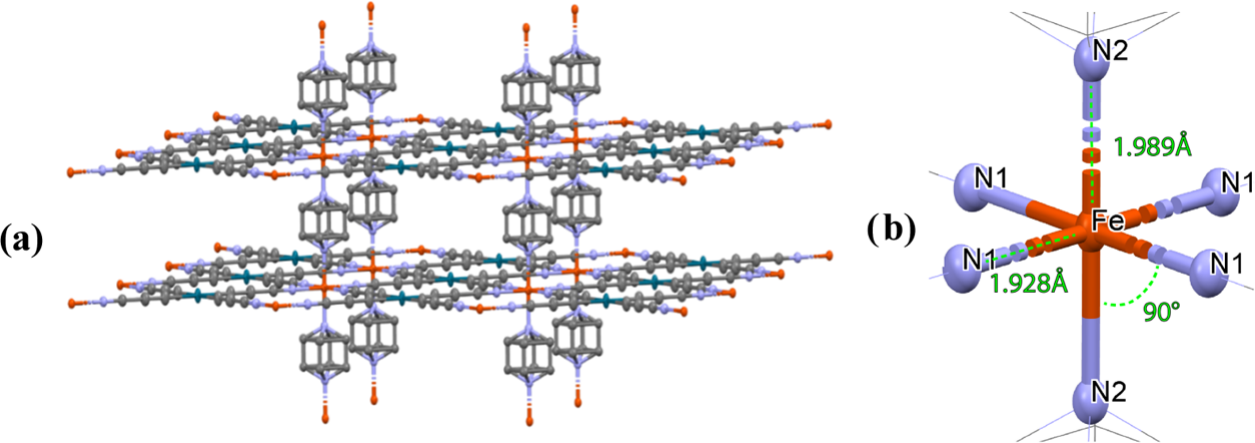
(a) Perspective view of **10** with disordered pyrazine molecules; hydrogens are omitted for clarity. (b) Detail on the Fe center with selected bond lengths and angles. Color code: orange = Fe, gray = C, blue = N, and petrol = Pd.

As expected, the structure is formed by alternate octahedral [FeN_6_]^2+^ cations and square-planar [Pd(C_3_N)_4_]^2–^ anions. Four equivalent [Pd(C_3_N)_4_]^2–^ groups coordinate via the N atoms at the equatorial positions of the octahedron, thus acting as bridges linking four Fe atoms and generating {Fe[Pd(C_3_N)_4_]}∞ layers. The layers lie on top of each other and are stacked by pyrazine molecules, which occupy the axial position of the octahedron, generating an open 3D framework. The octahedral coordination geometry around Fe(II) presents no distortions, due to the site symmetry 4/*mmm* all angles between cis-ligands are 90°. The Fe–N distances are 1.928 Å for the Fe–N1 bonds (equatorial) and 1.989 Å for the Fe–N2 bonds (axial), which are in agreement with typical Fe–N bond lengths for Fe(II) complexes in the LS state. The structure of **10** is depicted in Figure 1.

**Figure 2:**
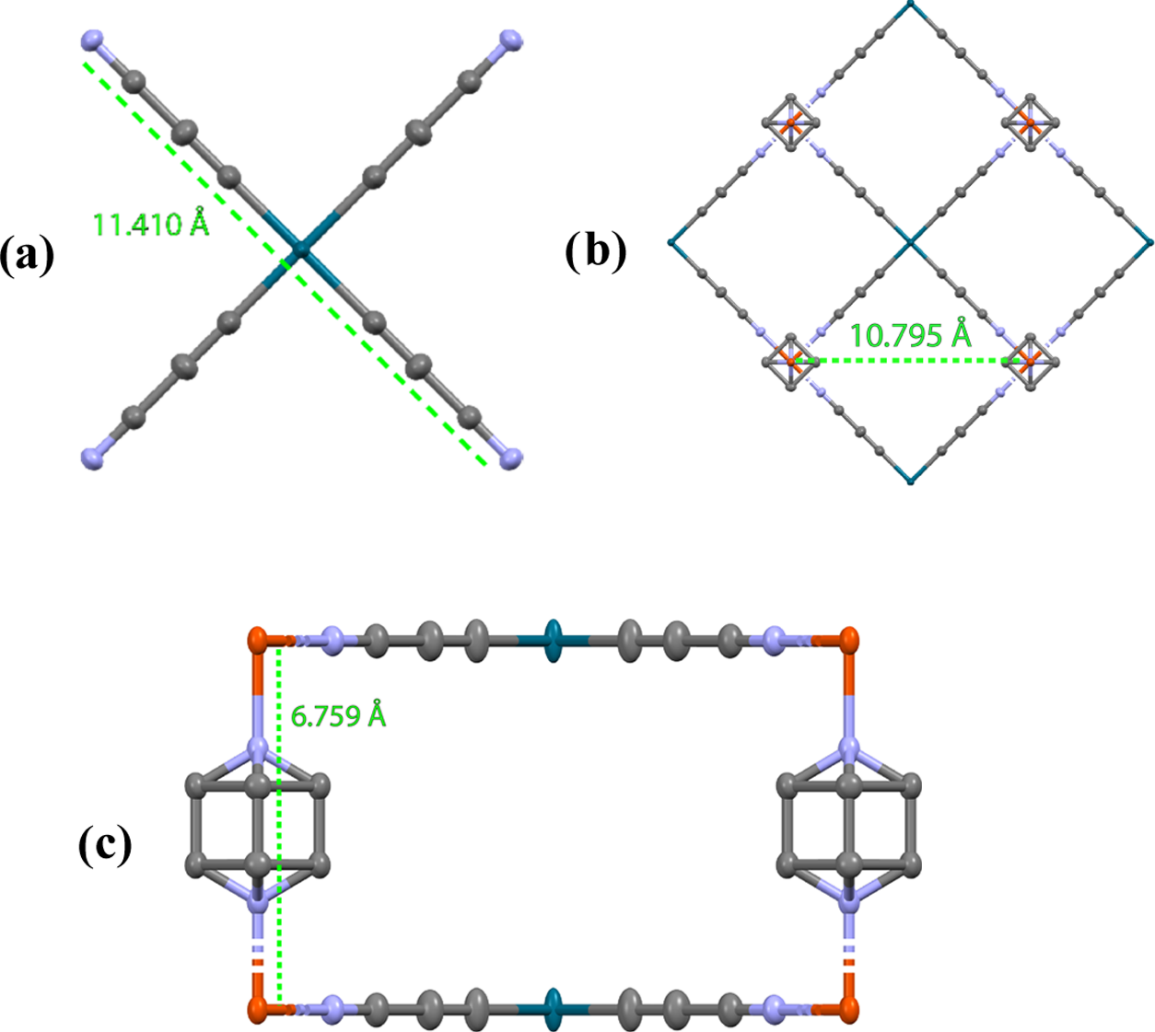
(a) N–N distance in the [Pd(C3N)4]^2–^ fragment, viewed along the *c-*axis. (b) Fe–Fe distance on the *ab* plane, viewed along the *c*-axis. (c) Fe–Fe distance between layers, viewed along the *a*-axis. Hydrogens have been omitted for clarity. Color code: orange = Fe, gray = C, blue = N, and petrol = Pd.

Comparison of **10** with **pzNi** and **pzPt** (no deposited structure for **pzPd** could be found) gives a good sense of the increase in the guest-accessible volume for this second generation of spin-switchable Hofmann-type networks: the N–N distances in the linker are 6.029 and 6.279 Å for [Ni(CN)_4_]^2–^ and [Pt(CN)_4_]^2–^, respectively, whereas in [Pd(C_3_N)_4_]^2–^, it is almost double, namely, 11.410 Å. As a result, the Fe–Fe distance in the {Fe[Pd(C_3_N)_4_]}∞ layers (which then determines the size of the pores) increases from 6.911/7.114 Å in **pzNi** and 7.184 Å in **pzPt** to 10.795 Å in **10**. The Fe–Fe distance between different layers remains practically the same, namely, 6.780 and 6.783 Å in **pzNi** and **pzPt**, respectively, vs 6.759 Å in **10** (see Figure 2).

**Figure 3:**
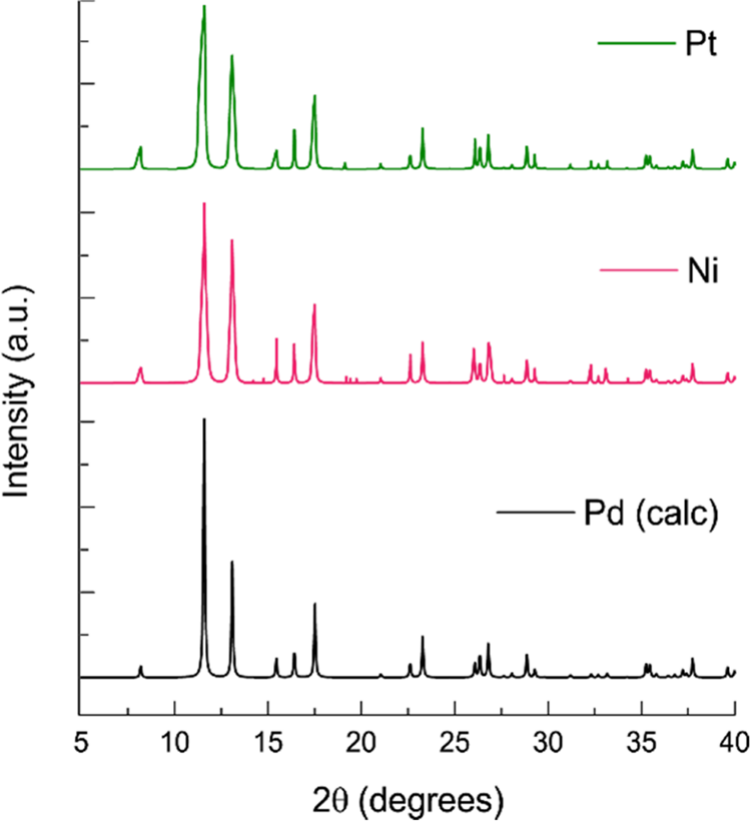
PXRD diffractogram of compounds **9** and **11** measured at 200 K and the calculated pattern for **10**.

The increase in the linkers’ length is reflected in the volume of guest-accessible voids (determined with Platon^33^) and the resulting porosity of the PCPs. With a guest-accessible volume of 454 Å^3^, compound **10** presents a 5-fold increase compared to the reference cyanide-based systems, for which guest-accessible voids have a volume of 94 Å^3^ (**pzNi**) and 83.6 Å^3^ (**pzPt**).^23,24^ It is also worth comparing compound **10** with other reported expanded Hofmann-type SCO-PCPs. For example, an elongation on the *c*-axis using bpac instead of pz in a cyanide-based compound results in guest-accessible voids with a volume of 293.6 Å^3^, although the axial Fe–Fe distance is doubled compared to the pz analogue (13.662 Å).^25^ Moreover, **10** shows a drastic increase in porosity: the voids in compound **10** constitute 57.7% of the unit cell’s volume, whereas the value for Fe(bpac)[Pt(CN)_4_]^25^ is 41.7% and only 23.9% for Fe(pz)[Pt(CN)_4_].^23^ Similar values for the volume of guest-accessible voids are found in Fe(bpeben)[Pt(CN)_4_] with 511 Å^3^, but in this case they constitute only 48.9% of the cell’s volume.^26^ Powder X-ray diffraction patterns for the structural confirmation of compounds **9** and **11** are depicted in Figure 3.

### Magnetic Characterization and Guest-Dependent Magnetic Behavior

**Figure 4:**
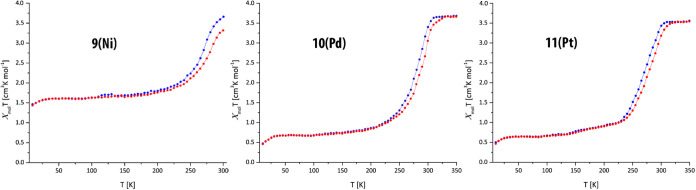
Temperature-dependent magnetic susceptibility of compounds **9–11**; red = heating, blue = cooling.

Temperature-dependent magnetic susceptibility was measured for compounds **9**, **10**, and **11** in temperature ranges 10–305 K (**9**) and 10–350 K (**10**, **11**). Due to the significant temperature sensitivity of **9**, magnetic measurements of the PCP guest composites were not performed for this compound because the resulting data would be incomplete and thus would not provide additional information. The PCPs exhibit a different behavior based on the presence/absence and nature of guest molecules. Compound **9** undergoes a gradual and incomplete spin transition (the LS state is not fully reached) with hysteresis and shows χ_mol_*T* values between 1.5 and 3.7 cm^3^ K mol^–1^, typical for Fe(II) in the HS state. Compounds **10** and **11** show a gradual but complete spin transition with a 10 K hysteresis and χ_mol_*T* values between 0.5 and 3.6 cm^3^ K mol^–1^, again typical values for Fe(II). *T*↑ values are 285 and 275 K and *T*↓ values are 275 and 265 K for **10** and **11**, respectively. Results are depicted in Figure 4. It must be mentioned that the measurements were performed on the material obtained after the synthesis; considering its amorphous nature, the observed magnetic behavior is remarkable, especially for the presence of a small hysteresis.

**Figure 5:**
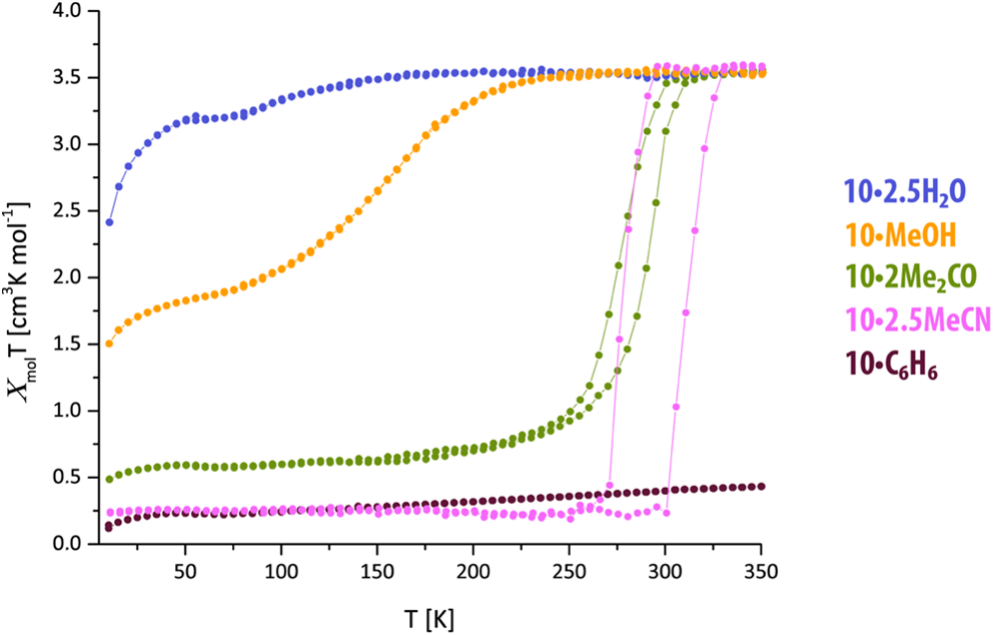
Temperature-dependent magnetic susceptibility of **10·guest**.

Guest absorption causes a drastic change in the magnetic behavior, which is also dependent on the nature of the guest. An uptake of MeCN results in a complete and abrupt spin transition with a larger hysteresis for both **10** and **11**. *T*↑ is 315 and 300 K and *T*↓ is 280 and 285 K for **10** and **11**, respectively. In both cases, the guest improves the quality of the spin transition and broadens the hysteresis, which especially for **10** is noteworthy. The same type of behavior is observed when the PCPs adsorb acetone, although the hysteresis is not as large as for MeCN. Absorption of MeOH results in a gradual and incomplete spin transition without hysteresis. As observed for other compounds of this class, certain guest molecules induce the stabilization of one spin state: for **10** and **11**, water stabilizes the HS state at all temperatures, whereas benzene stabilizes the LS state at all temperatures. Although magnetic measurements were not carried out, this still seems to be valid for **9** as well: when exposed to water, the powder maintained a bright yellow color even after cooling in liquid nitrogen, and when exposed to benzene, it immediately turned from yellow to deep red, and the color was maintained after heating (up to 35 °C). Results are listed in Figure 5.

Comparison of these results with the ones reported in the literature for **pzM** highlights some differences between the two families of compounds. The empty **pzM** networks show a rather abrupt spin transition with a larger hysteresis, in the range 20–30 K.^22−24^ The difference in abruptness and width of hysteresis has probably two reasons: the additional flexibility provided by the longer C_3_N fragment, resulting in a decrease of cooperativity, and the fact that **pzM** precipitates as microcrystalline powders, whereas **9**, **10**, and **11** are amorphous. The PCP guest composites also show a different behavior compared to that of **pzM·guest**. The two main factors influencing the magnetic behavior of the PCP guest composites are guest-sized and host–guest interactions. In **pzM**, benzene absorption stabilizes the HS state at all temperatures, thus showing an opposite behavior as that of **9**–**11**. The reason for this different behavior lies with all probability in the different pore size: **10**, **11**, and **pzM** all absorb one molecule of benzene per Fe center, and in **pzM**, the benzene ring is large enough to prevent a shrinkage, thus causing the stabilization of the HS state, whereas for **10** and **11**, it is reasonable to assume that π–π interactions between bz and pz prevail and stabilize the system in the LS state. The stabilization of the HS state by water is presumably of dual nature: water weakens the ligand field strength of Fe(II), and also the small H_2_O molecules completely fill the voids, providing a rigidity that stabilizes the HS state.^24,34^

### Bidirectional Chemo-Switching and Memory Effect

**Scheme 2:**
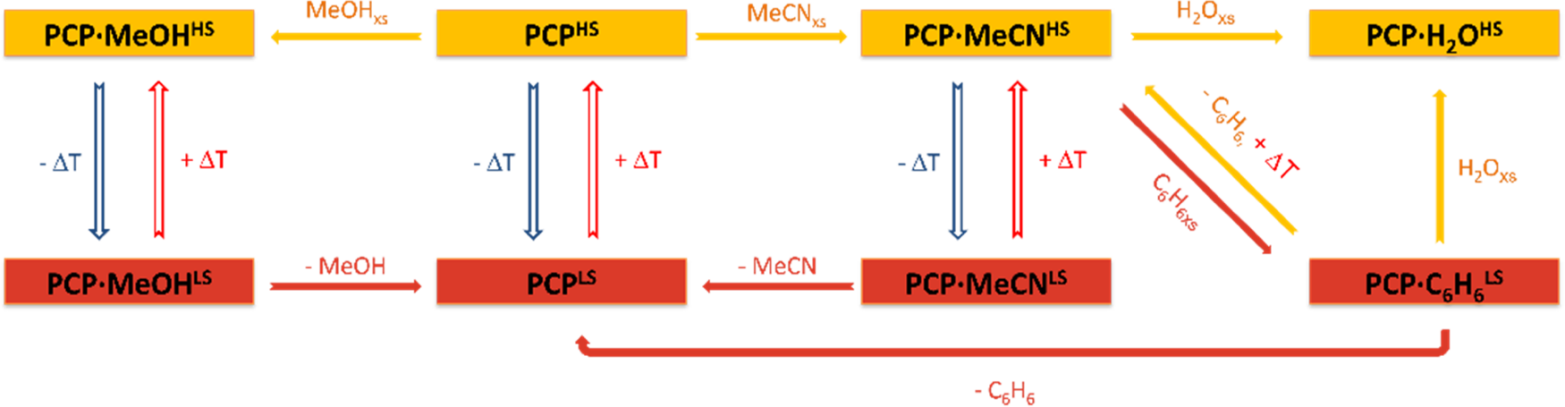
Schematic Representation of the In Situ Bidirectional Chemo-Switching and Memory Effect of Compounds **9–11**

In situ bidirectional chemo-switching of the spin state and memory effect were observed for all PCPs, confirming a synergistic behavior between guest absorption/desorption and spin transition as in **pzM**. A schematic illustration of the results is depicted in Scheme 2.

Exposure of the PCPs to MeCN at room temperature yields PCP·MeCN^HS^. Lowering the temperature triggers the spin transition to PCP·MeCN^LS^, as already shown by magnetic susceptibility measurements. Exposing PCP·MeCN^HS^ to an excess of MeOH results in the replacement of MeCN with MeOH, and again, lowering the temperature brings PCP·MeOH in the LS state (despite the incomplete transition, a color change could be observed). After exposing both guest-free PCPs and PCP·MeCN^HS^ to benzene without changing the temperature, a chemo-switching is observed, giving PCP·C_6_H_6_^LS^. Moreover, the PCPs show a memory effect: exposure of PCP·C_**6**_H_**6**_^LS^ to MeCN at room temperature leads to PCP·MeCN^LS^. The LS state is also retained after benzene desorption, yielding PCP^LS^. Exposing PCP·C_**6**_H_**6**_^LS^ to water results in another chemo-switching to PCP·H_2_O^HS^.

### Infrared Spectroscopy

Fourier transform infrared-mid-infrared (FTIR-MIR) spectra were measured for all of the reported compounds; variable temperature measurements were recorded for compounds **9**–**11** to monitor changes in the IR absorption bands upon spin transition. Compound **4** shows an intense and broad ν_NH_2__ absorption band with two peaks (typical for primary amides) located at 3286 and 3178 cm^–1^, the ν_C≡C_ band at 2109 cm^–1^ and the ν_C=O_ band at 1645 cm^–1^. Compound **5** presents the ν_CH_ of the butyl groups in the range 2957–2854 cm^–1^ and the ν_CN_ at 2247 cm^–1^. Compound **6**–**8** show the ν_CH_ of the ethyl groups in the range 2980–2990 cm^–1^, the ν_CN_ band at 2203, 2214, and 2209 cm^–1^, and the ν_C≡C_ band at 2038, 2049, and 2050 cm^–1^ for **6**, **7** and **8**, respectively, which are in accordance with literature-reported data.^31^ Coordination to Fe(II) causes a shift of the absorption bands in **9**–**11**, and for these compounds, a shift is also observed by changing the measurement temperature since it triggers the spin transition. Results are reported in Table 2, for the spectra see the Supporting Information.

**Table 1:** Cystallographic Parameters for **10** Described in the Small Unit Cell

	**10**
formula	C_16_H_4_FeN_6_Pd
weight [g mol^–1^]	442.5
*T* [K]	200
color	red
shape	block
crystal system	tetragonal
space group	*P*4*/mmm*
*a* [Å]	10.7946(15)
*c* [Å]	6.7591(14)
*V* [Å^3^]	787.6(3)
*z*	1
ρcalc [g cm^–3^]	0.933
μ [mm^–1^]	1.035
measured refl’s	12722
unique refl’s	522
F(000)	214
rint	0.0616
GooF	1.169
*R*_1_	0.0582
*wR*_2_	0.1893
no. of parameters	32
CCDC	2366166

**Figure 6:**
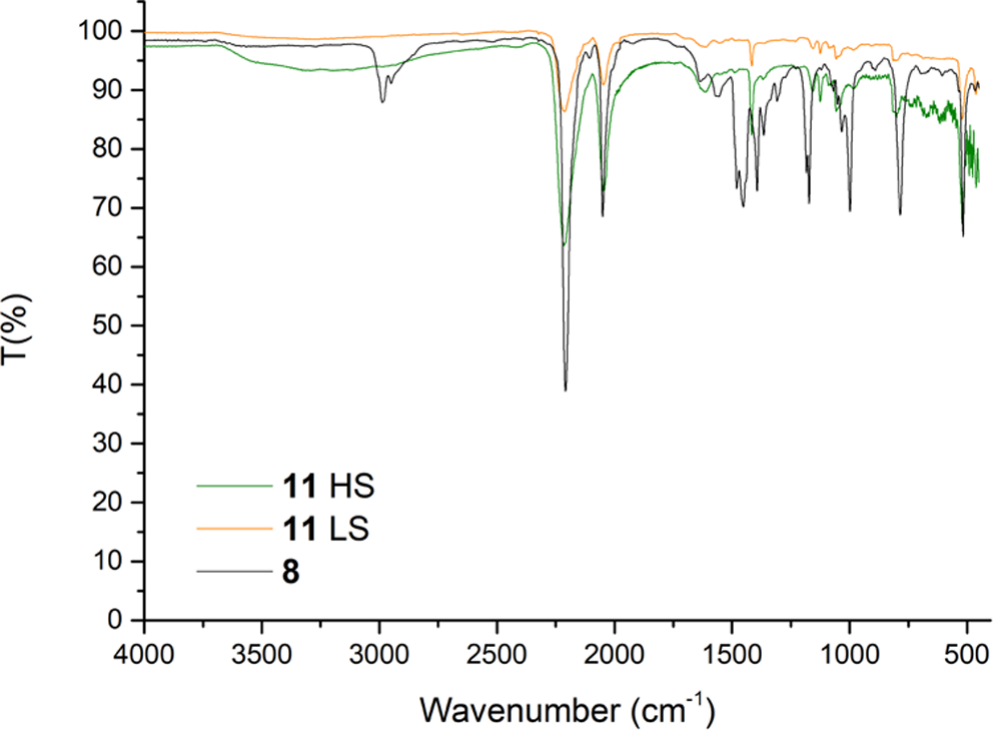
MIR spectra comparison of **8**, **11HS**, and **11LS**.

For **6**–**8**, the C≡C bands shift to a higher wavenumber with increasing stability of the M–C bond. As expected, the ν_CN_ band is affected by coordination to Fe(II), and in **9**–**11**, it shifts to higher wavenumbers compared to **6**–**8**. A band broadening is also observed for both bands, which is characteristic for successful coordination. In **9**–**11**, a small shift of the ν_CN_ band is also observed upon spin transition with lower wavenumbers for the HS state due to the increase in the Fe–N bond length. A comparison of the IR spectra of **8**, **11HS**, and **11LS** is depicted in Figure 6.

**Table 2:** IR Absorption Bands for Compounds **6–11**

IR mode	**6** (cm^–1^)	**7** (cm^–1^)	**8** (cm^–1^)
ν_CN_	2203	2208	2214
ν_C≡C_	2038	2049	2050
ν_M-C_	517	516	517

	**9** (cm^–1^)		**10** (cm^–1^)		**11** (cm^–1^)	
IR mode	HS	LS	HS	LS	HS	LS
ν_CN_	2215	2220	2223	2218	2214	2212
ν_C≡C_	2038	2038	2050	2053	2048	2049

## Conclusions

The target compounds {Fe(pz)[M^II^(C_3_N)_4_]} (M = Ni, Pd, or Pt) were successfully synthesized and characterized in their structure, magnetic properties, and host–guest chemistry interactions. The newly developed synthetic method to produce Fe[M(C_3_N)_4_]∞ layers proved to be successful, and for the first time, an elongation of the networks within the equatorial plane was performed without recurring to aromatic (multi)ring systems or linear M^I^CN_2_ linkers. As a result, the desired regular 3D structure of the **pzM** analogues was retained without network interpenetration. The addition of an acetylenic unit to the CN fragment was confirmed to be an advantageous strategy because due to the linearity and low steric hindrance of the C_3_N fragment, the elongation by just two carbon atoms results in a drastic increase of both the volume of guest-accessible voids and the porosity of the resulting PCPs. Elongation along the *c*-axis with longer pillar ligands is not as effective since these are based on multiring systems which are bulky and take away part of the pores’ space. Consequently, extremely long ligands are needed to reach the same characteristics of the void volume and porosity of **9**–**11**, but this often results in a poor magnetic behavior and/or ligand incorporation. Another drawback is that enlarging the pores only along the *c*-axis can still prevent the uptake of larger guests since they are not necessarily flat and rod-shaped. Most importantly, compounds **9**–**11** retained the desired SCO properties, showing hysteretic SCO as well as a guest-dependent magnetic behavior, in situ bidirectional chemo-switching of the spin state, and the memory effect, two properties that make this class of compounds particularly attractive. A negative aspect is of course the sensitivity of **9** toward temperature, which makes it very difficult to handle. As mentioned in the “Synthesis” paragraph, some synthetic aspects need to be improved by finding new reaction pathways to obtain species containing the [M(C_3_N)_4_]^2–^ fragment without recurring the use of highly toxic trialkyl-Sn^IV^ species. Another hindering feature is the amorphous nature of the materials and the consequent difficulty of growing single crystals. Different methods were tried out (solvent diffusion, layering techniques, slow reaction diffusion, hydrothermal crystallization, nonaqueous gel diffusion), but none worked. Since all of these methods entail a fast introduction of Fe^2+^, thinking of alternative methods with slow Fe^2+^ diffusion led to electrocrystallization, which finally was successful. Slow diffusion of Fe^2+^ seems to be a crucial step for crystal growth. A layering method with extremely diluted solutions was fortunately good enough to obtain crystalline powders, but despite countless attempts, no single crystals suitable for measurement could be grown with this method. Nonetheless, with this work, we showed the proof of principle for the synthesis of the materials and we believe to have paved the way for a new approach to Hofmann-type SCO-PCPs. The present work is a starting point for the further improvement of this new family of Hofmann-type SCO-PCPs. The next steps include ulterior extension of the frameworks, both in the *ab* plane through additional C≡C subunits and on the *c*-axis with longer pillar ligands. Lastly, functionalization of the pillar ligands will be an important step because it could allow for a selective guest absorption of large and functional guests.

## Experimental Section

### Methodology

All operations involving Fe(II) were carried out under an inert gas atmosphere (argon 5.0). The glassware was oven-dried at 120 °C before use for at least 2 h. All solvents for the complexation reactions were dried before use and stored over a 3 Å molecular sieve under argon.^35^ Unless otherwise stated, all starting materials were commercially obtained and used without further purification. All NMR spectra were recorded in dry deuterated solvents on a Bruker Avance UltraShield 400 MHz. Chemical shifts are reported in parts per million; ^1^H and ^13^C shifts are referenced against the residual solvent resonance. The magnetic moment of the Fe(II) complexes was measured using a physical property measurement system (PPMS) by Quantum Design. The experimental setup consisted of a vibrating sample magnetometer attachment (VSM), bearing a brass sample holder with a quartz-glass powder container. The magnetic moment was determined in an external field of 1 T in the range of 10–350 K. Quantification of the guest uptake was made by NMR measurements, dissolving the samples in DMSO and using the peak of pyrazine as the reference. Room- and variable- temperature midrange (4000–450 cm^–1^) infrared spectra were recorded by the attenuated total reflection (ATR) technique on a PerkinElmer Spectrum 400 system, fitted with a coolable/heatable PIKE GladiATR unit.^36^ Single crystals attached to a glass fiber by using perfluorinated oil were examined at 200 K on a Bruker KAPPA APEX II diffractometer equipped with a CCD detector with Mo Kα radiation (Incoatec Microfocus Source IμS: 30 W, multilayer mirror, λ = 0.71073 Å) and an Oxford Cryosystems Cryostream 800 Plus LT device. Data handling with integration and absorption correction by evaluation of multiscans was done with the Bruker Apex5 suite.^37^ The structures were solved by direct methods;^38^ subsequent difference Fourier syntheses and least-squares refinements yielded the positions of the remaining atoms using SHELXL software^39^ implemented in the shelXle GUI tool.^40^ For the iron(II) complexes, protons were placed at calculated positions and refined as riding on the parent C atoms. All non-H atoms were refined with anisotropic displacement parameters. The refinement of the crystal structure in the averaged small unit cell in the space group *P*4/*mmm* achieved *R* values (*R*_1_ = 0.058, *wR*_2_ = 0.189) as compared to (*R*_1_ = 0.085, *wR*_2_ = 0.286) for the superstructure in the space group *P*4/*mbm*. Apart from the specific arrangement of the Pz molecules, there were no significant differences in the general atomic arrangement as well as interatomic bond distances when comparing the two structure models. The powder X-ray diffraction measurements were carried out on a PANalytical X’Pert Pro diffractometer in Bragg–Brentano geometry using Cu Kα1,2 radiation filtered with a BBHD mirror and an X’Celerator linear detector. For in situ experiments below ambient temperature, an Oxford PheniX cryochamber from Oxford Cryosystems was used. The powder samples were mounted on an Anton Paar domed sample holder. The actual sample temperature was directly monitored using a thermocouple on the sample holder. The diffractograms were evaluated by using the PANalytical program suite HighScorePlus, correcting for the background. For the single-crystal growth via electrocrystallization, an ISO-TECH IPS1810H DC laboratory power supply was used. Thermal analysis was performed using a Netzsch STA 449 C Jupiter device in the temperature range 25–500 °C.

### Single-Crystal Growth

**Figure 7:**
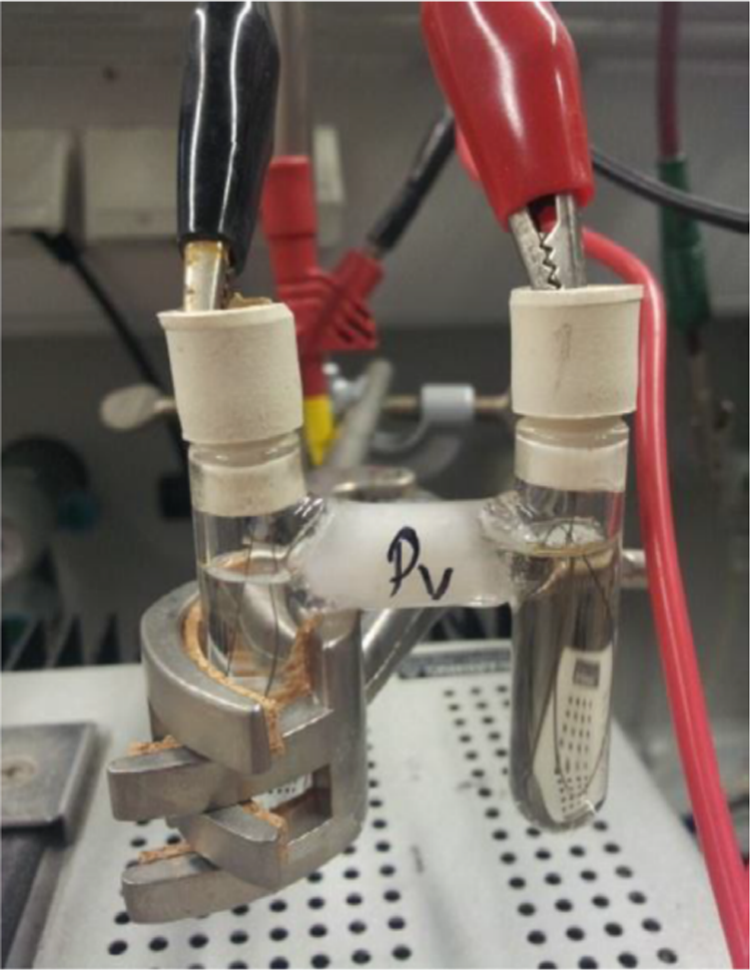
Experimental setup for single-crystal growth via electrocrystallization.

The experimental setup for the crystal growth consisted of an H-tube with a frit loaded with a MeCN solution of (*n*-Bu)_4_N(BF_4_) on one side (cathode) and a MeCN solution of pyrazine and **7** (after filtration on activated charcoal) on the other side (anode). An Fe wire was introduced on both sides and the H-tube was closed with two septa. A voltage of 1.5 V was applied between the two iron wires causing the slow anodic dissolution of the Fe wire. Square-shaped, dark red single crystals formed on the glass wall’s anodic side. The setup is depicted in Figure 7.

### Synthesis

#### Synthesis of Tetraethylammoniumtetrachloronickelate (**1**)

Nickel(II) chloride hexahydrate (10 g, 1 equiv) and tetraethylammonium chloride (14.01 g, 2.01 equiv) were suspended in 50 mL of EtOH and stirred for 18 h at RT. The bright green solution was concentrated until crystallization started and cooled to 0 °C. The precipitated turquoise solid was separated, washed with cold CH_2_Cl_2_, and dried under vacuum (11.1 g, 79.8% yield).

#### Synthesis of Tetraethylammoniumtetrachloropalladate (**2**)

Palladium(II) chloride (1.5 g, 1 equiv) and tetraethylammonium chloride (2.8 g, 2 equiv) were suspended in 20 mL of MeNO_2_ and heated for 18 h at 75 °C. The solvent was evaporated, and the dark red residue was triturated in 10 mL of CH_2_Cl_2_, cooled to 0 °C, and filtrated. The solid was dried under vacuum, and the product was isolated as a dark red powder (3.9 g, 90.7% yield).

#### Synthesis of Tetraethylammoniumtetrachloroplatinate (**3**)

Platinum(II) chloride (1.25 g, 1 equiv) and tetraethylammonium chloride (1.56 g, 2 equiv) were suspended in 20 mL of MeNO_2_ and heated for 18 h at 75 °C. The solvent was evaporated, and the dark red residue triturated in 10 mL of CH_2_Cl_2_, cooled to 0 °C, and filtrated. The solid was dried under vacuum, and the product was isolated as a dark red/brown powder (2.5 g, 90% yield).

#### Synthesis of 2-Propynamide (**4**)

A 250 mL single-neck round-bottom flask was cooled to −50 °C. Gaseous NH_3_ was insufflated through a septum and condensed in the flask. Methylpropiolate (10 g, 1 equiv) was added dropwise, and the reaction was left stirring at −40 °C for 4 h. After quick warming under a N_2_ stream, Et_2_O was added to the orange solution, the precipitated solid was separated, and the solvent was removed under reduced pressure yielding the product as an orange solid (quantitative yield).
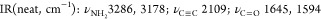

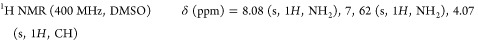


#### Synthesis of 3-(Tributylstannyl)propiolonitrile (**5**)

The synthesis was conducted under an inert atmosphere with a standard Schlenk technique. A 100 mL Schlenk flask was charged with bis(tributyltin) oxide (31.16 g, 1 equiv), dry benzene (20 mL), and 3 Å molecular sieves and cooled to −196 °C. The flask was connected via a glass tube to a 500 mL Schlenk flask charged with **4** (8.21 g, 0.12 mol, 2 equiv). The system was evacuated and filled with Ar three times, respectively, and finally, P_2_O_5_ (50.6 g, 6 equiv) was added in the 500 mL flask under an Ar stream. The system was evacuated to 1 mbar and kept under vacuum for the whole reaction time. The reaction mixture was heated to 200 °C with a heating mantle, and a vigorous reaction was observed, and the formation of large amounts of vapor indicated the formation of 2-propynenitrile. This was accompanied by a foaming of the pentoxide mixture. After approximately 15 min, the dehydration of the amide was complete (no further gas evolution was observed), and the reaction mixture turned to a black tar. The system was vented with Ar and the solid product was left to warm up to room temperature and further stirred for 10 min. All solids were filtered off and the crude product (dark brown liquid) was distilled under reduced pressure (bp 110 °C at 0.7 mbar) to isolate the product as a colorless liquid with a pungent smell (19 g, 94% yield).
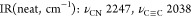

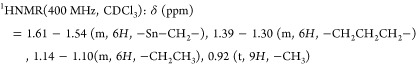

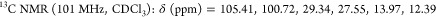

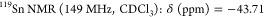


#### Synthesis of Tetraethylammonium Tetrakis(cyanoethynyl)nickelate (**6**)

The reaction was conducted under an inert atmosphere with the standard Schlenk technique. **1** (2.5 g, 1 equiv) was dissolved in 25 mL of dry MeCN and the solution was cooled to 0 °C. **5** (11.07 g, 6 equiv) was added under cooling, and the solution was left stirring for 18 h. The mixture was filtered with a pressure filter to separate the solid deposit, and the filtrate was poured in dry Et_2_O (ca. 200 mL). A precipitate formed, and it was again separated via pressure filtration and washed 3 times with small portions of Et_2_O. The solid was finally dried under vacuum to isolate the product as a dark gray solid (1.5 g, 53.3% yield).
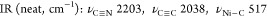


#### Synthesis of Tetraethylammonium Tetrakis(cyanoethynyl)palladate (**7**)

The same procedure as that for **6**, with 2 g (1 equiv) of **2** and 6 g (6 equiv) of **5**, was used. The product was a brown solid (1.2 g, 71.8% yield).
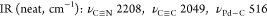


#### Synthesis of Tetraethylammonium Tetrakis(cyanoethynyl)platinate (**8**)

The same procedure as that for **6**, with 2 g (1 equiv) of **3** and 6.83 g (6 equiv) of **5**, was used. The product was a brown solid (1.7 g, 86.5% yield).
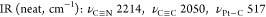


#### Synthesis of Anhydrous Fe(BF_4_)_2_

A three-neck Schlenk flask was charged with reduced iron powder in 40 mL of tetrahydrofuran (THF) and equipped with a reflux condenser and a septum. HBF_4_·Et_2_O was added dropwise and the mixture was heated to reflux and left stirring for 5 days (until all iron powder was consumed). The mixture consisting of the solvent and undissolved Fe(BF_4_)_2_ was then cooled in an ice bath to let the solid settle, and the solvent was removed with a syringe. The solid was washed three times with dry Et_2_O and dried under vacuum to isolate the product as a white powder (quantitative yield).

#### Synthesis of {[Fe(pz)][Ni(C_3_N)_4_]} (**9**)

A microwave vial was charged with 2 mL of dry MeCN, pyrazine (4.63 mg, 1 equiv), and Fe(BF_4_)_2_ (13.25 mg, 1 equiv). Thirty mg (0.06 mmol, 1 equiv) of **6** was dissolved in 2 mL of dry MeCN and the solution was filtered over activated charcoal in a Pasteur pipet directly into the vial. Immediate precipitation of a yellow powder was observed. The suspension was centrifuged, the solvent was removed with a syringe, and the solid was dried under vacuum. The product was isolated as a yellow-orange powder (15.1 mg, 65.8% yield).
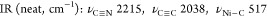


#### Synthesis of {[Fe(pz)][Pd(C_3_N)_4_]} (**10**)

The same procedure as that for **9**, with 4.24 mg (1 equiv) pyrazine, 12.14 mg (1 equiv) of Fe(BF_4_)_2_, and 30 mg (0.053 mmol, 1 equiv) of **7**, was used. The product was isolated as a yellow-orange powder (18 mg, 76.9% yield).
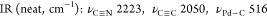


#### Synthesis of {[Fe(pz)][Pt(C_3_N)_4_]} (**11**)

The same procedure as that for **9**, with 3.66 mg (1 equiv) pyrazine, 10.5 mg (1 equiv) of Fe(BF_4_)_2_, and 30 mg (0.046 mmol, 1 equiv) of **8**, was used. The product was isolated as an orange powder (21.3 mg, 87.6% yield).
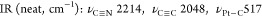


## Supporting Information Available

IR spectra of compounds **4**–**11**; NMR spectra of compounds **4** and **5;** temperature-dependent magnetic susceptibility plots of **11·guest**; and crystallographic parameters for compound **10** in the large cell (PDF)
