# Supplementary material for: Tetrakis-Cyanoacetylides as Building Blocks for a Second Generation of Spin-Switchable Hofmann-type Networks with Enhanced Porosity
Source: Inorg Chem. 2024 Sep 3;63(37):17067–76. doi: 10.1021/acs.inorgchem.4c02732 (PMC11409210; doi:10.1021/acs.inorgchem.4c02732)
Supplement: Supplementary file 1 — ic4c02732_si_001.pdf [file ic4c02732_si_001.pdf]

SUPPORTING INFORMATION

Tetrakis-cyanoacetylides as building block for a second generation of spin-switchable Hofmann type-networks with enhanced porosity

Willi Zeni[a], Danny Müller[a], Werner Artner[b], Gerald Giester[c], Michael Reissner[d], Peter Weinberger[a]**

[a] DI Willi Zeni, Dr. Danny Müller, Associate-Prof. Dr. Peter Weinberger
Institute of Applied Synthetic Chemistry, TU Wien
Getreidemarkt 9/163-01-3, 1060 Vienna, Austria

willi.zeni@tuwien.ac.at
peter.e163.weinberger@tuwien.ac.at

[b] Dipl.- Ing. Werner Artner
X-Ray Center, TU Wien, Getreidemarkt 9/057-4, 1060 Vienna, Austria

[c] Ao.Univ.Prof. Dr. Gerald Giester
Department of Mineralogy and Crystallography
University of Vienna, Josef-Holaubek-Platz 2, 1090 Vienna, Austria

[d] Ao.Univ.Prof. Dr. Michael Reissner
Institute of Solid State Physics, TU Wien
Wiedner Hauptstraße 8-10/138, 1040 Vienna, Austria

INFRARED SPECTRA OF COMPOUNDS 4-11

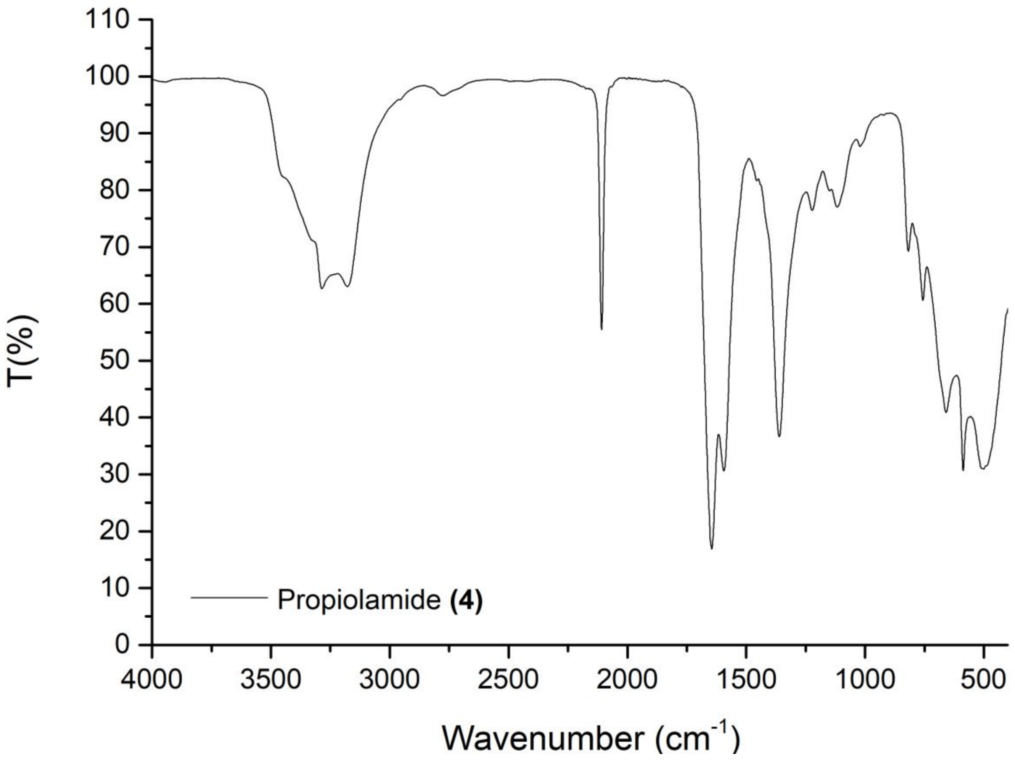

*Figure S1: FT-IR-MIR spectrum of propiolamide (**4**)*

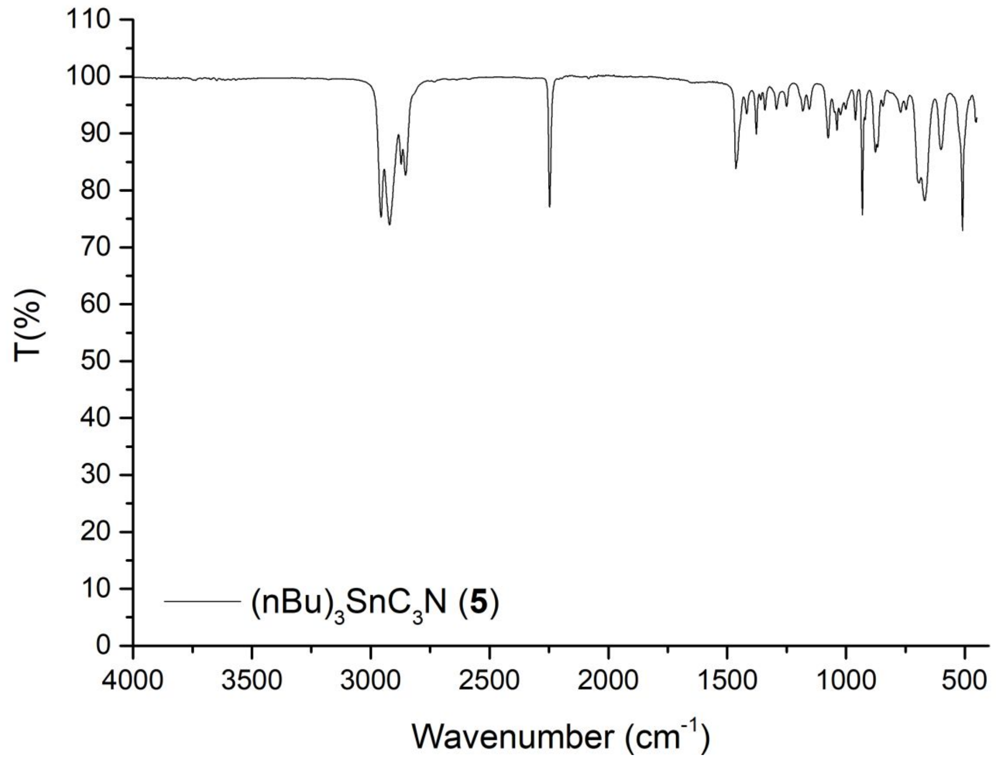

*Figure S2: FT-IR-MIR spectrum of 3-(trisbutylstannil)propiolonitrile (**5**)*

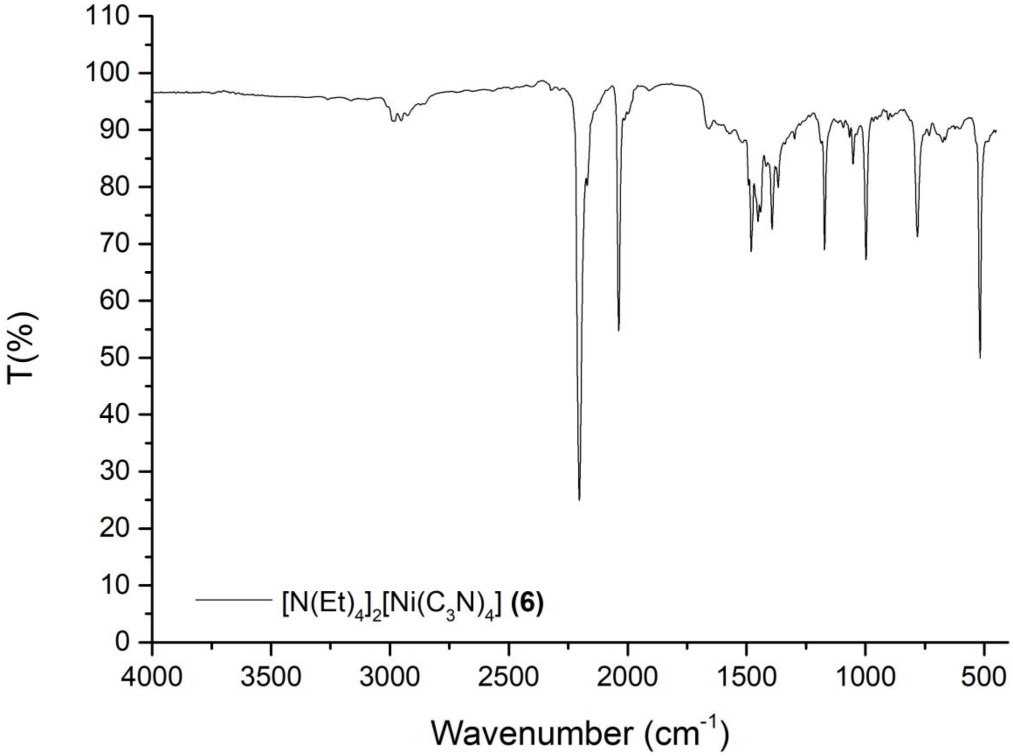

*Figure S3: FT-IR-MIR spectrum of tetraethylammonium tetrakis(cyanoethynil) nickelate (**6**)*

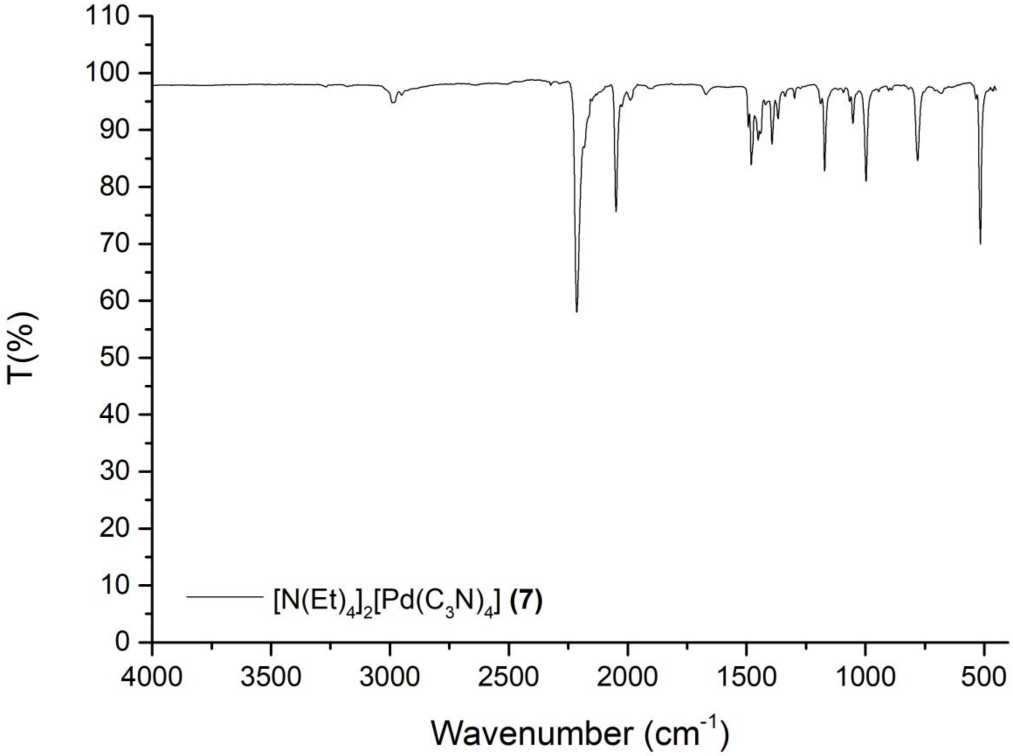

*Figure S4: FT-IR-MIR spectrum of tetraethylammonium tetrakis(cyanoethynil) palladate (**7**)*

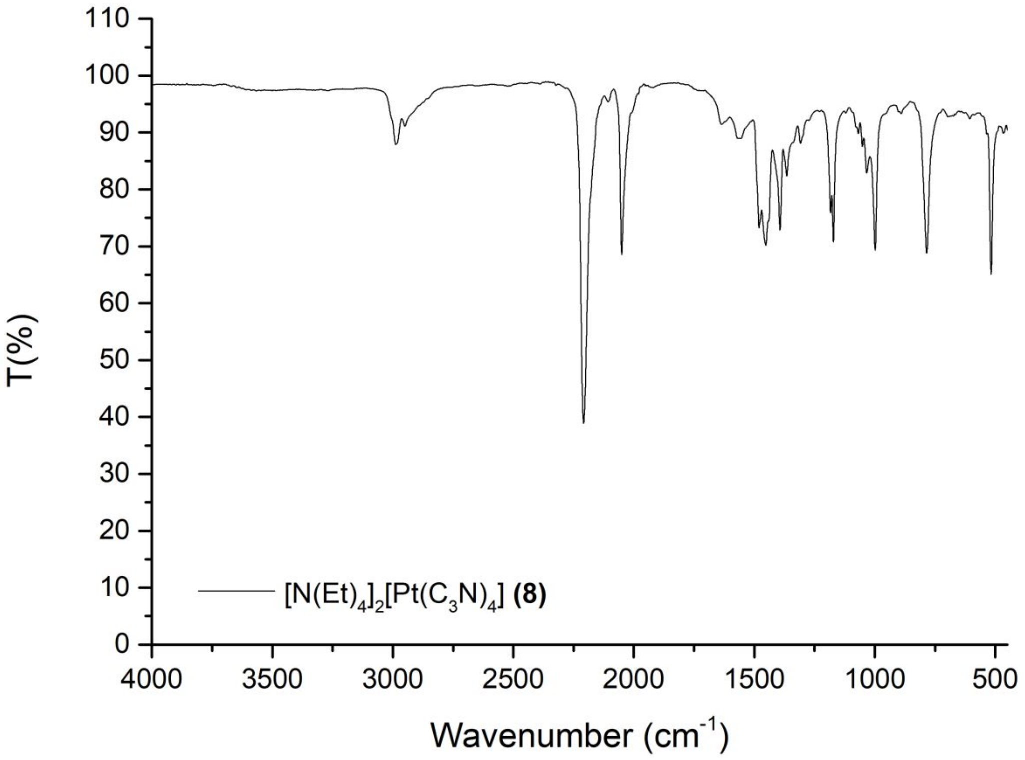

*Figure S5: FT-IR-MIR spectrum of tetraethylammonium tetrakis(cyanoethynil) platinate (**8**)*

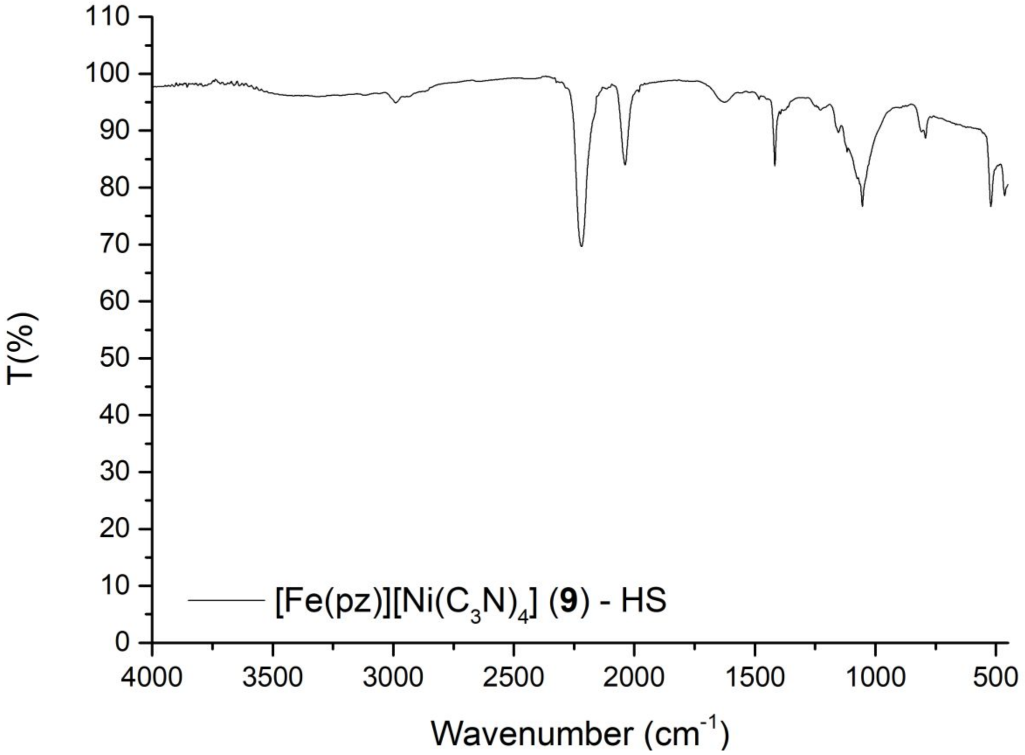

*Figure S6: FT-IR-MIR spectrum of [Fe(pz)][Ni(C_3N)$_4$] - HS (**9**)*

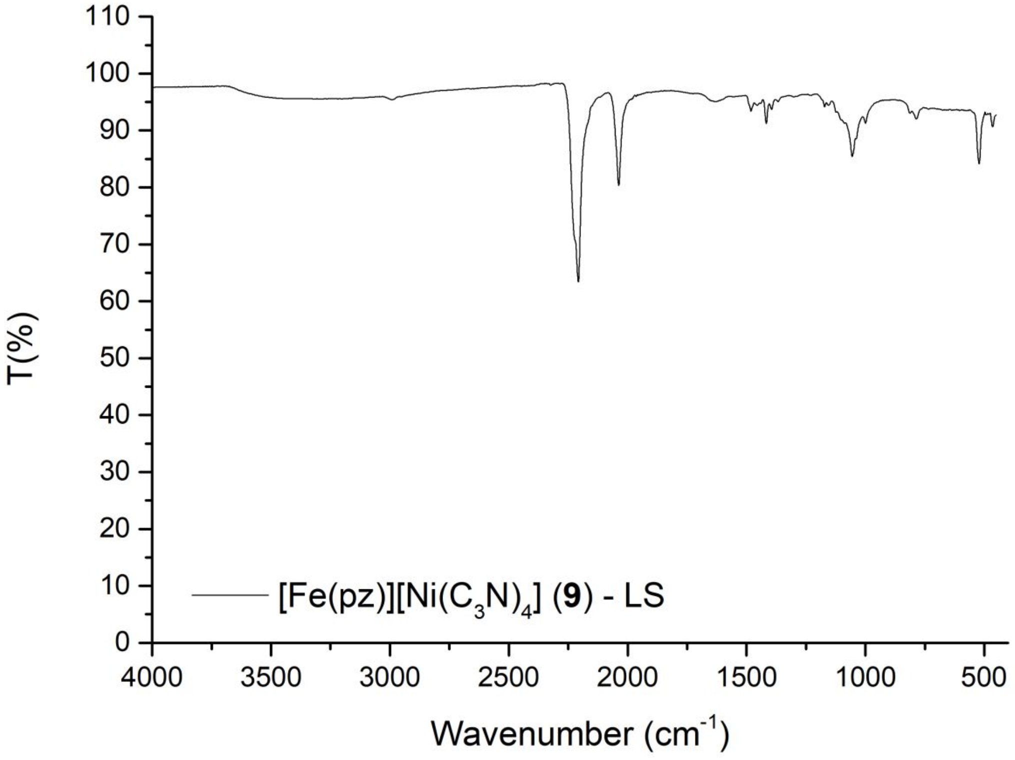

*Figure S7: FT-IR-MIR spectrum of [Fe(pz)][Ni(C_3N)$_4$] - LS (**9**)*

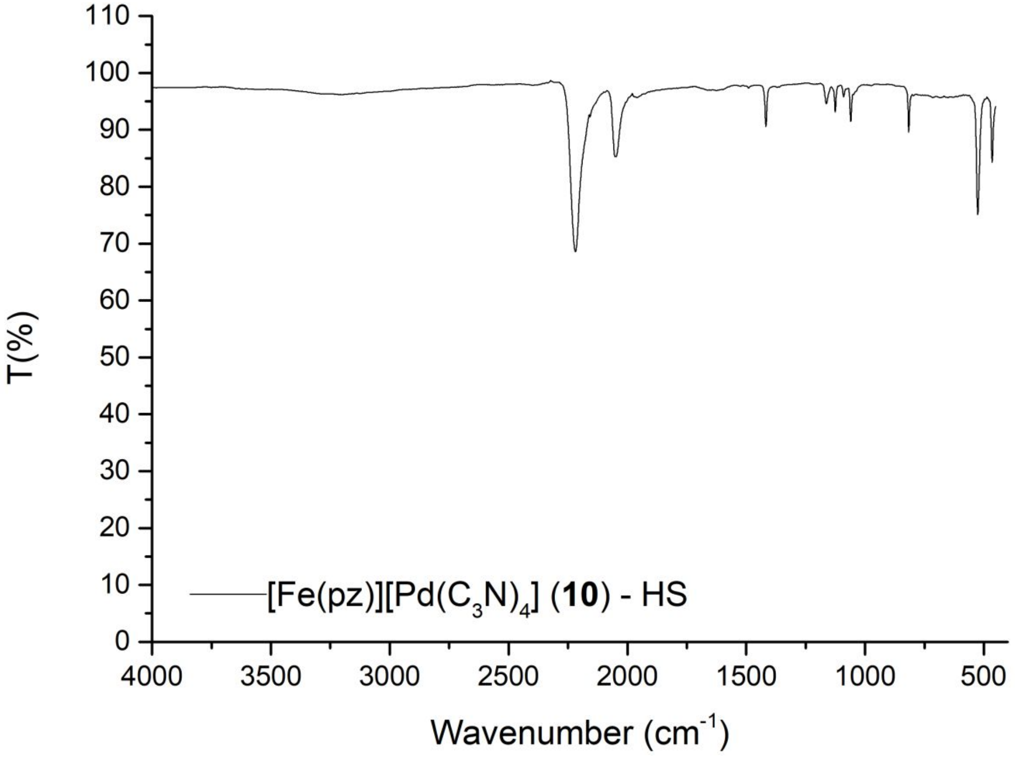

*Figure S8: FT-IR-MIR spectrum of [Fe(pz)][Pd(C_3N)$_4$] - HS (**10**)*

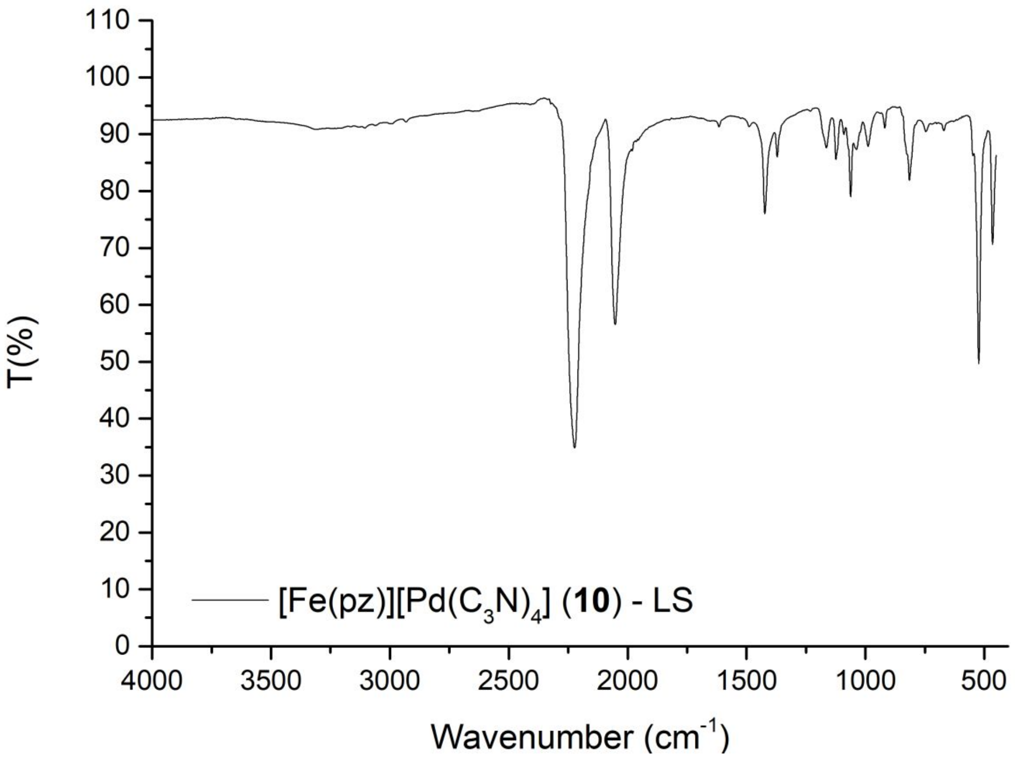

*Figure S9: FT-IR-MIR spectrum of [Fe(pz)][Pd(C_3N)$_4$] - LS (**10**)*

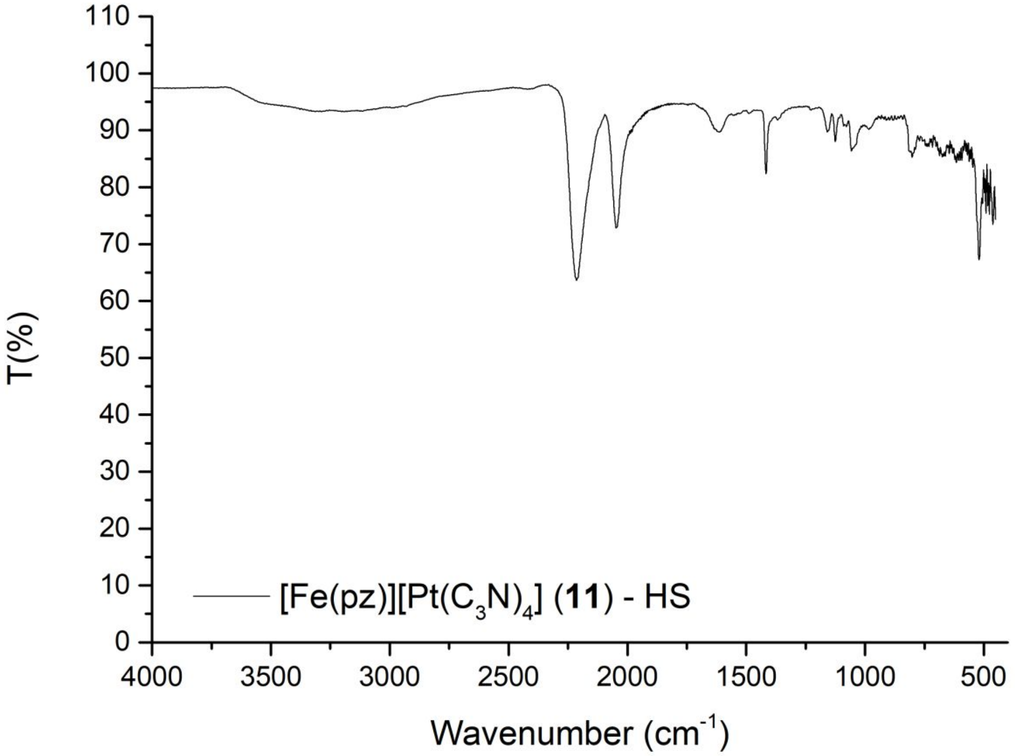

*Figure S10: FT-IR-MIR spectrum of [Fe(pz)][Pt(C_3N)$_4$] - HS (**11**)*

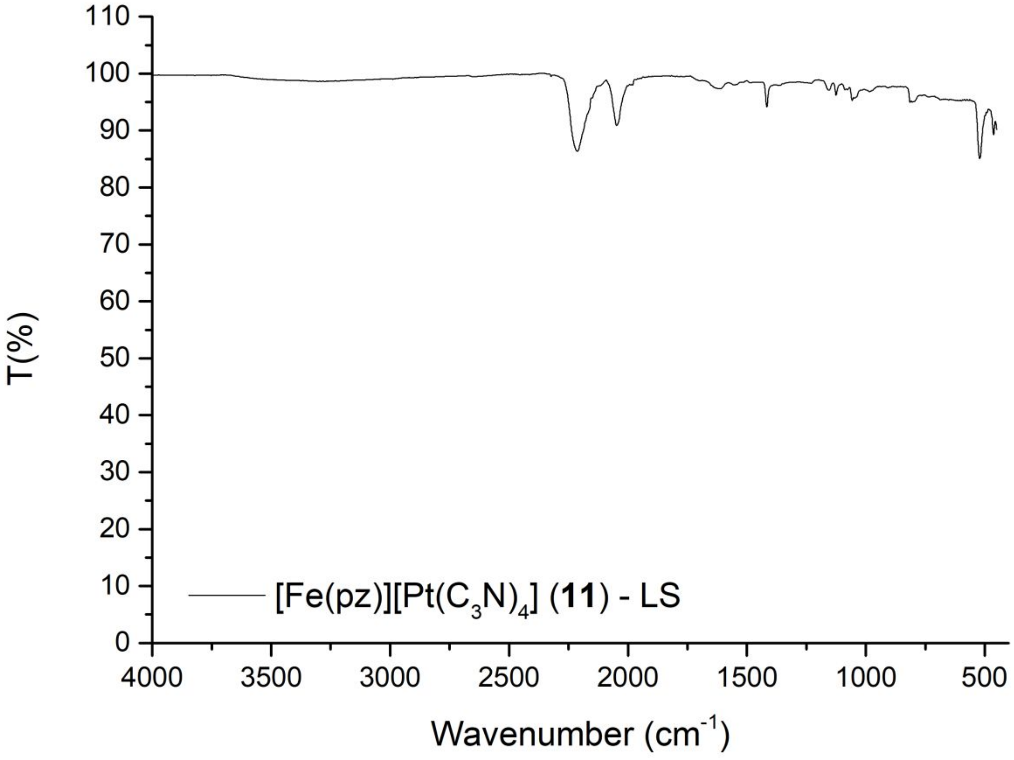

*Figure S11: FT-IR-MIR spectrum of [Fe(pz)][Pt(C_3N)$_4$] - LS (**11**)*

TEMPERATURE DEPENDENT MAGNETIC SUSCEPTIBILITY OF PCP•GUEST COMPOSITES

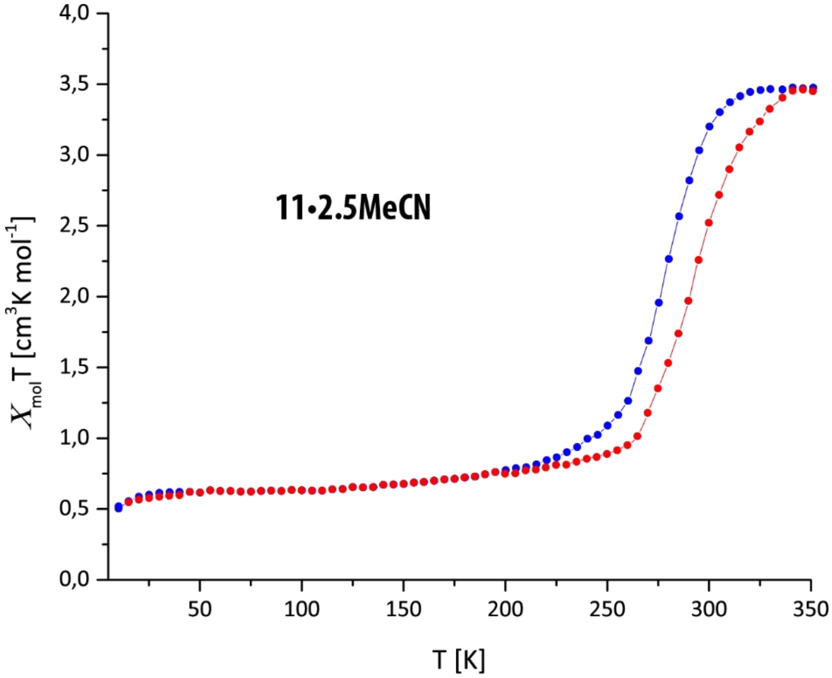

*Figure S12: Temperature dependent magnetic susceptibility of **11·2.5MeCN***

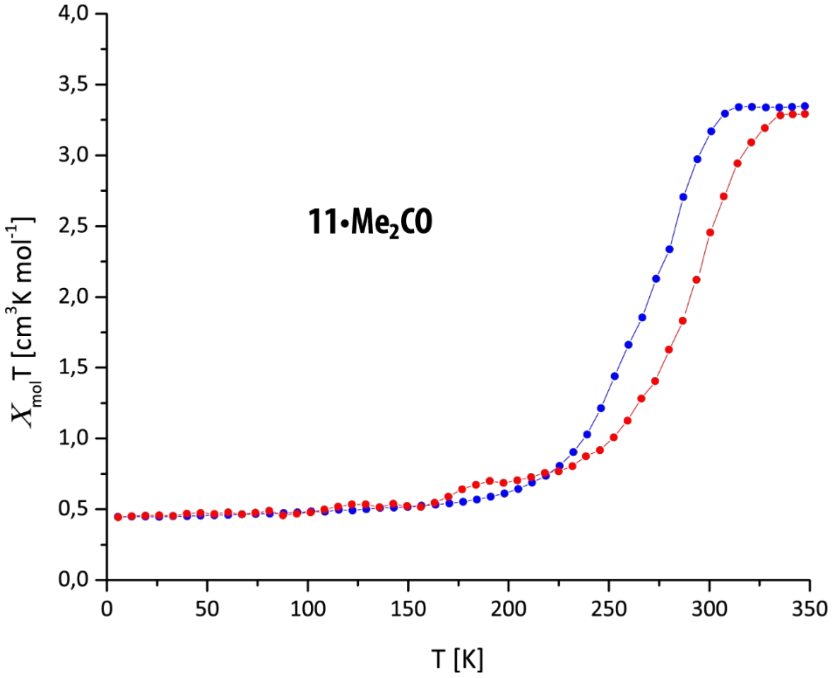

*Figure S13: Temperature dependent magnetic susceptibility of **11·Me$_2$CO***

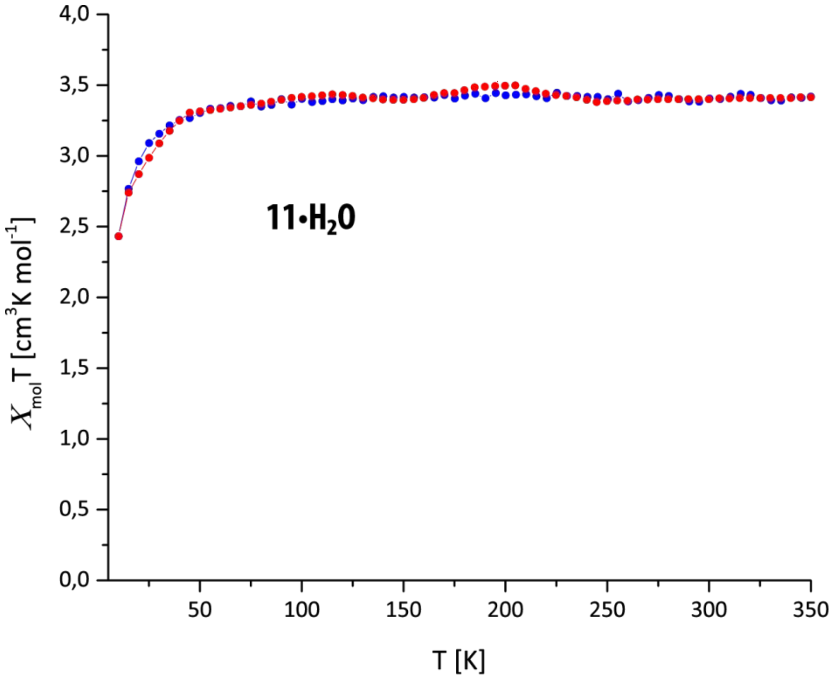

Figure S14: Temperature dependent magnetic susceptibility of ***11·H₂O***

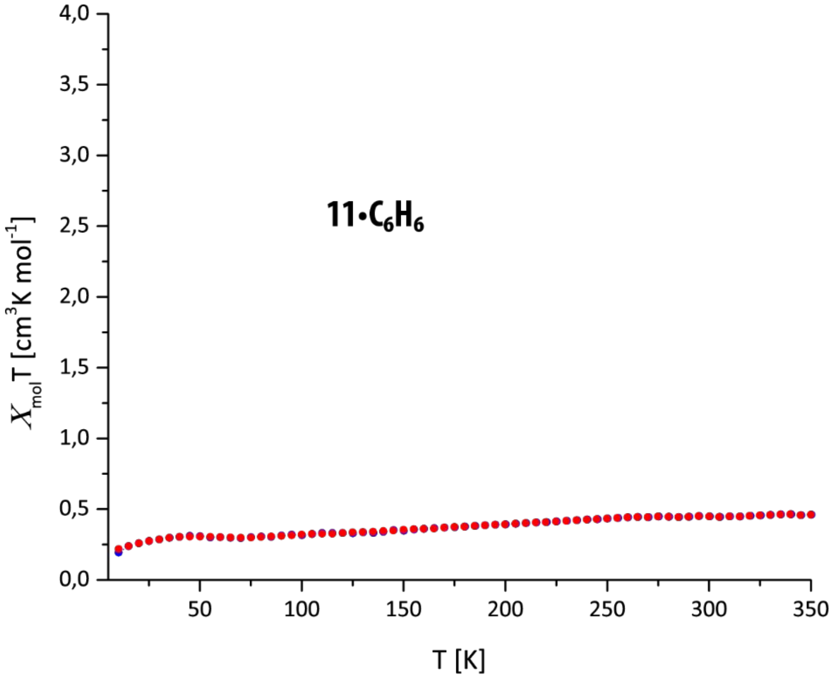

Figure S15: Temperature dependent magnetic susceptibility of ***11·C_6H_6***

SELECTED BOND-LENGTHS FOR 10 – SMALL CELL

Table S1: Selected bond-lengths for 10 in the small unit cell

Atom1	Atom2	Length (Å)
Pd	C3	1.982
Fe	N1	1.928
Fe	N2	1.99
N1	C1	1.137
C1	C2	1.38
C2	C3	1.21

SUPERSTRUCTURE-SPACE GROUP *P4/mbm*

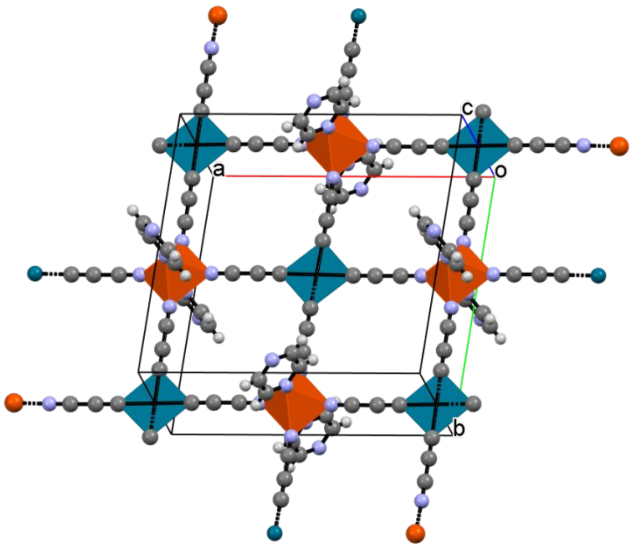

*Figure S16: Superstructure of **10** in the space group P4/mbm with specific arrangement of the pz rings. The coordination figures around Fe and Pd are indicated with orange and petrol color, respectively. Grey =C, blue=N, white=H*

Table S2: Crystallographic parameters for 10 in the large unit cell

	**10**
Formula	$C_{16}H_4FeN_6Pd$
Weight [g mol-1]	442.5
T [K]	200
Colour	red
Shape	block
Crystal System	tetragonal
Space Group	*P4/mbm*
a [Å]	15.266 (3)
c [Å]	6.759(2)
V [Å³]	1575.2
z	2
ρcalc. [g cm-3]	0.933
μ [mm-1]	1.035
Measured Refl's.	24045
Unique Refl's	926
F(000)	428
Rint	0.0919
GooF	1.131
R1	0.0855
wR2	0.2859
No. of parameters	41
CCDC	2366167

Table S3: Selected bond-lengths for 10 in the large unit cell

Atom1	Atom2	Length
Pd	C3	1.984
Fe	N1	1.93
Fe	N2	1.986
N1	C1	1.137
C1	C2	1.374
C2	C3	1.209

THERMAL ANALYSIS

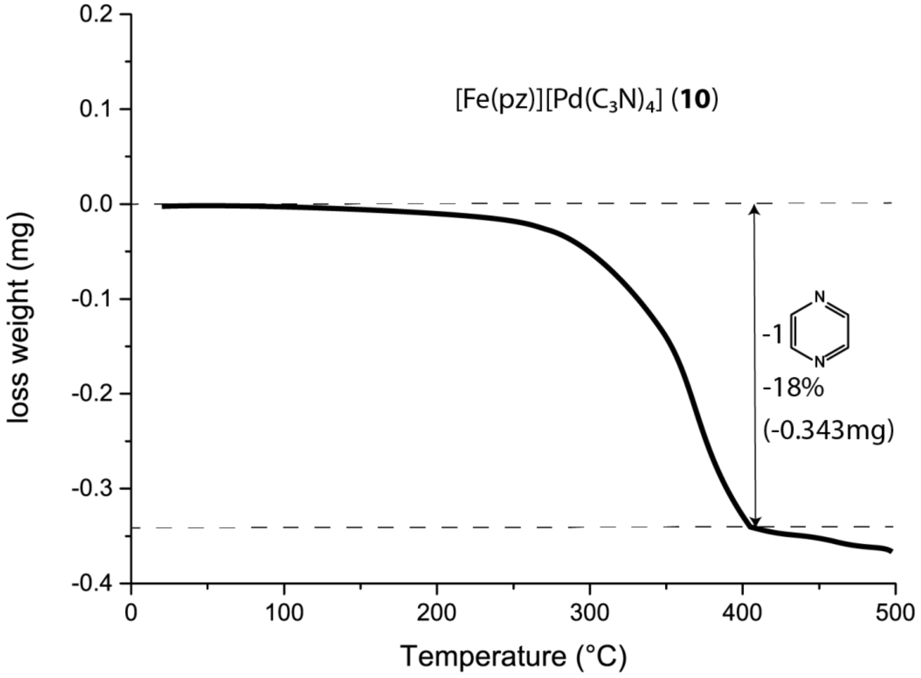

*Figure S17: Thermal analysis for **10***

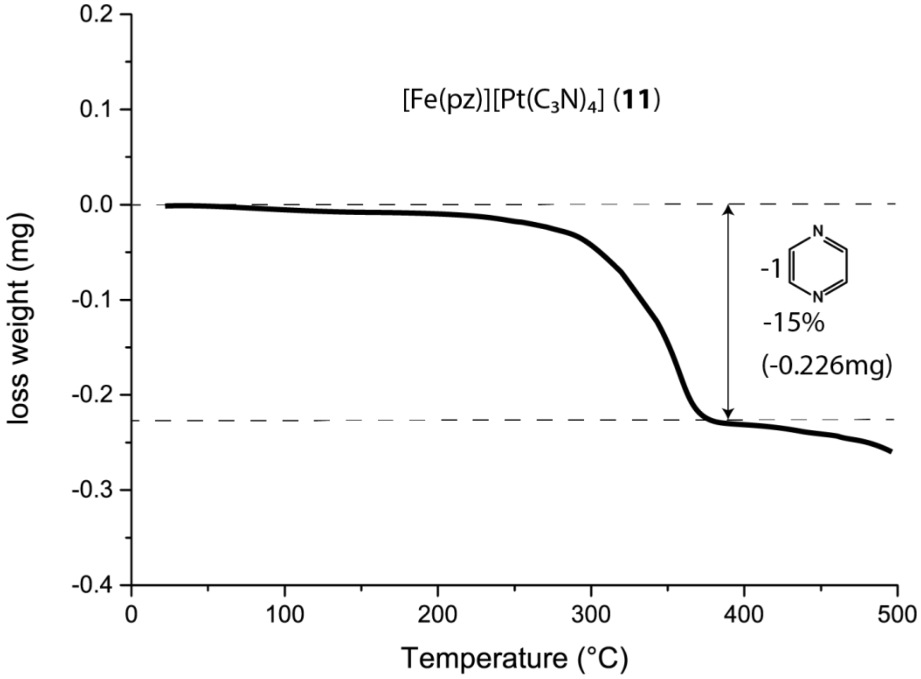

*Figure S18: Thermal analysis for **11***

NMR SPECTRA OF COMPOUNDS 4-5

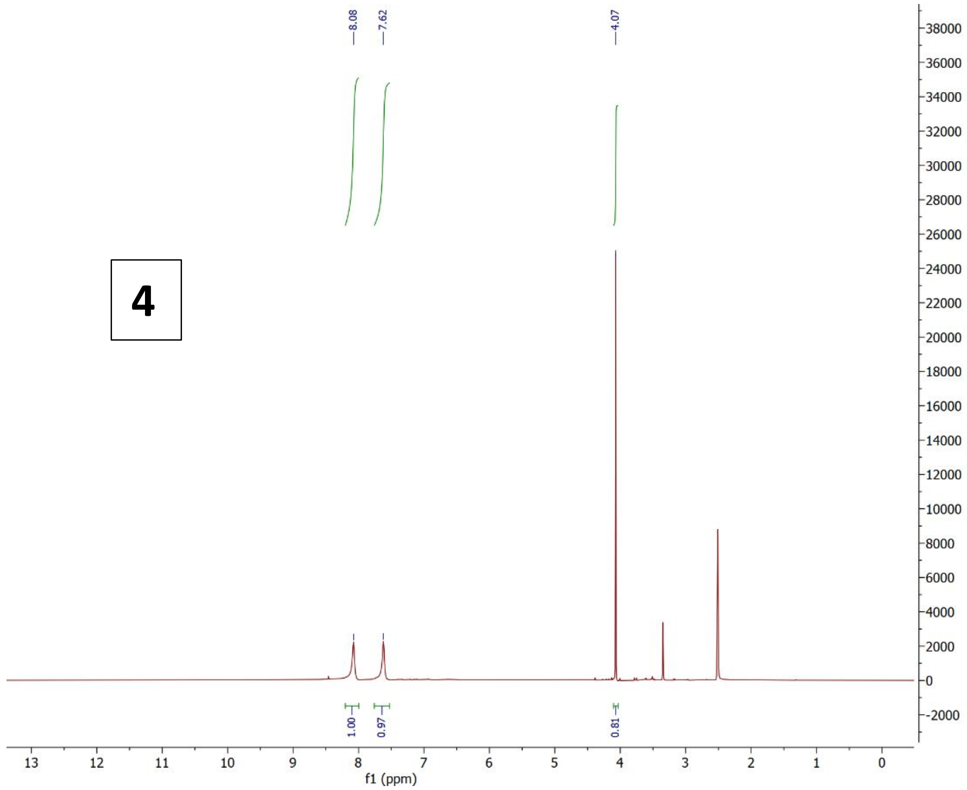

*Figure S19: ^{1}H NMR spectrum of propiolamide (**4**)*

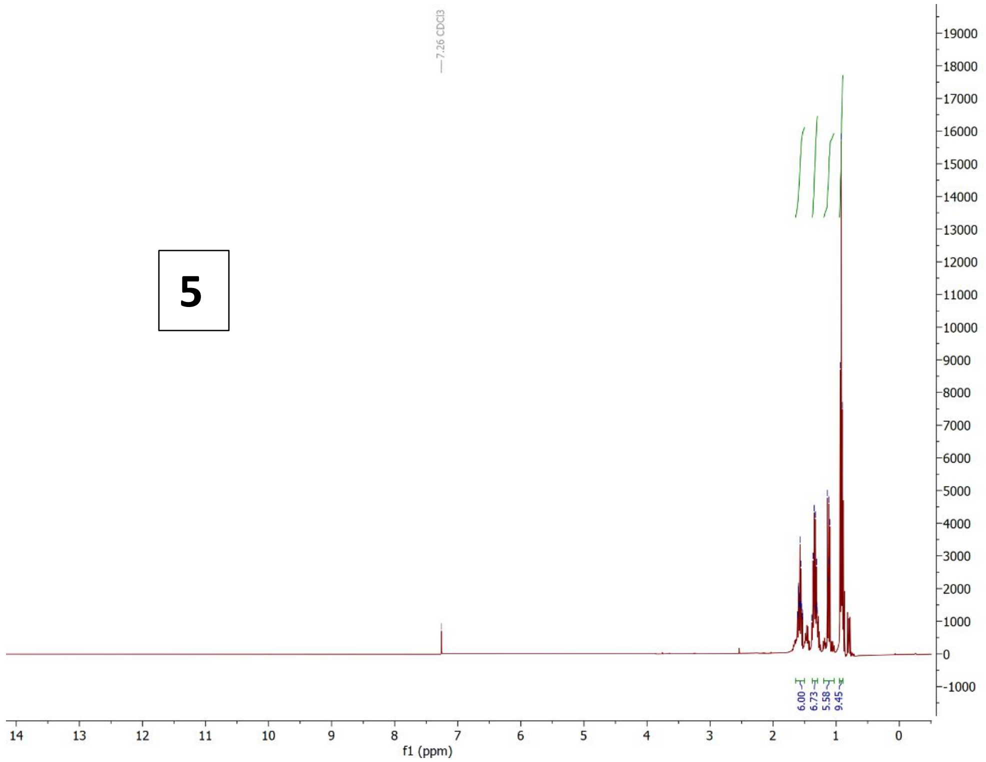

*Figure S20: ^{1}H NMR spectrum of 3-(trisbutylstannil)propiolonitrile (**5**)*
